# Association between pet ownership and physical activity levels, atopic conditions, and mental health in Singapore: a propensity score-matched analysis

**DOI:** 10.1038/s41598-020-76739-2

**Published:** 2020-11-16

**Authors:** Ying Xian Goh, Joel Shi Quan Tan, Nicholas L. Syn, Beverley Shu Wen Tan, Jia Ying Low, Yi Han Foo, Waikit Fung, Brandon Yi Da Hoong, Junxiong Pang, Phase IV CHP 2020 Group 8, Qi Xuan Lim, Jieying Wee, Terence Yan Ming Ng, Hsin Han Elisha Chow, Yu Ling Ng, Jiamin Charmaine Chong, Charmaine Yan Yeo, Lorraine Hui En Tan, Abigail E Xuan Sim, Ahmad bin Hanifah Marican Abdurrahman, Carissa-Jill Yinn Soon, Ian Jun Yan Wee, Julia Yu Xin Ng, Xin Chen Lim, Lloyd Jee Hean Ng, Mervin Nathan Han Hui Lim, Wei Ren Ong, Wen Tao Daniel Ong, Ryan Gabriel Tan, S. Hema Viganeshwari, Santhosh S/O Sasidaran Pillai, Shawn Soon Han Chan, Siti Humaira Bte Mohd Kamil, Isabel Soh, Mengyue Su, Yu Xiang Tan, Valerie Tian Wei Chew, Lily Wei Yun Yang, Mun Yike Fiona Yee

**Affiliations:** 1 Yong Loo Lin School of Medicine, National University of Singapore and National University Health System, Singapore, Singapore; 2 Saw Swee Hock School of Public Health, National University of Singapore and National University Health System, 12 Science Drive 2, Level 10, Singapore, 117549 Singapore; 3 Centre for Infectious Disease Epidemiology and Research, National University of Singapore, Singapore, Singapore

**Keywords:** Public health, Quality of life, Risk factors, Cardiovascular diseases, Psychiatric disorders, Asthma

## Abstract

Although existing literature increasingly suggests a positive influence of pet ownership on human physical activity levels, results from many European, American, and Japanese studies have been inconsistent. How pet ownership impacts mental health and atopy is likewise controversial and whether distinct demographic subgroups experience differential effects is unclear. This cross-sectional study surveyed participants (*n* = 823) via a self-administered online questionnaire. Comparisons of outcomes between pet owners and non-pet owners with subgroup analyses were performed within a propensity score-matched subset (*n* = 566) of respondents. There were no differences in physical activity levels or mental health scores between pet owners and non-pet owners. In subgroup analyses, compared to non-pet owners, main pet caregivers reported 14.1 (95% CI 2.79–25.3) and 19.0 (95% CI 4.70–33.3) more minutes per week of moderate- and vigorous-intensity physical activity respectively and higher SF-36 emotional well-being (*β* = 2.7, 95% CI 0.100–5.32) and energy scores (*β* = 3.8, 95% CI 0.410–7.27). Age was a significant effect modifier of the association between pet ownership and emotional well-being, energy and social functioning scores, with greater scores above the ages of 39, 35 and 39 years old respectively (interaction *p* = 0.043, 0.044, 0.042). Finally, pet acquisition was associated with worsening of allergic rhinitis, while pet ownership cessation was associated with improvement of allergic rhinitis and eczema symptoms. To our knowledge, this is the first study addressing the public health impact of pet ownership in Southeast Asia and its findings add contextual nuance to suggest potential benefits derived from pet ownership.

## Introduction

Pet ownership has long been advocated to bring about many health benefits to their owners^1^. Of the many potential health outcomes, three of them—namely physical activity, mental health and atopy—are among the most pertinent, with conflicting results reported for the latter two. Furthermore, most of the prior literature originates from American, European or East Asian countries, but to our knowledge, there has been no previous study from Southeast Asia investigating the effect of pet ownership on physical activity, mental well-being and atopic conditions.

Based on a review of 81 studies, a recent American Heart Association (AHA) scientific statement acknowledged the positive effects of pet ownership (specifically dog ownership) on physical activity levels^2^. There is also evidence supporting the cumulative effects of pet ownership over a person’s lifetime, with greater benefits at an older age compared to the young^3,4^.

However, the impact of pet ownership on mental health remains an area of controversy. Several studies found improved mental health among pet owners—potentially attributable to increased social interactions^5–7^, reduction in stress and anxiety levels^8–13^—while some others suggest a potentiation of psychological distress^13^. These findings are contended by other studies showing that pet ownership could be associated with higher levels of psychoticism and major depression in the elderly^14^. The demands of pet ownership, such as financial costs and distress, can also negatively impact one’s mental health^8^.

Another dimension of interest is the effect of pet ownership on atopic conditions (asthma, allergic rhinitis and eczema). Whilst there are studies suggesting that pet ownership or animal exposure protects against the development of atopic conditions^15–18^ via allergen desensitisation, other studies claim that animals otherwise serve as sources of allergens^19^ that may result in higher rates of diagnosis or worsening of atopic conditions^20–24^.

There were approximately 824,600 registered pets in Singapore in 2016^25^, and pet ownership continues to grow rapidly. To our knowledge, there has not been a local study thus far investigating the association between pet ownership and its impact on physical activity, mental health and atopy in the Singapore adult population. Hence, this study was conducted to address these knowledge gaps.

## Materials and methods

### Study design

This cross-sectional study surveyed adults aged 21 to 64 years living in Singapore for the past 6 months via an online questionnaire. To reduce participant heterogeneity and potential confounding biases, those with physical disabilities amounting to requiring assistance in at least 1 activity of daily living (ADLs—dressing, eating, ambulating, transferring, toileting, maintaining hygiene) and those who own therapy or guide dogs were excluded from the study.

Participants were recruited over an 11-day period in February 2020 by invitation through posters placed at participating veterinary clinics across Singapore, broadcast messages by the research team on online messaging applications (e.g., WhatsApp, Telegram), posts and advertisements on social media platforms (e.g., Facebook, Instagram), electronic mail to NUS Saw Swee Hock School of Public Health (SSHSPH) staff and students, as well as word-of-mouth. Broadcast messages were sent by 37 investigators (NUS Medicine medical students) to at least 15 other individuals each, including (but not limited to) family, friends and acquaintances.

A Participant Information Sheet was provided at the start of the questionnaire, followed by three screening questions—for participants to acknowledge that neither they nor their household members had previously participated in the survey.

The online questionnaire was self-administered in one of four official languages (English, Chinese, Malay, Tamil). Data was collected anonymously via REDCap, a browser-based online questionnaire platform, and stored securely on the NUS SSHSPH server.

The study protocol was approved by the NUS SSHSPH Departmental Ethics Review Committee (DERC Reference Number SSHSPH-011), with waiver of written informed consent granted as all data collected was anonymous and did not contain personal identifiers. All methods were performed in accordance with the relevant guidelines and regulations.

### Definitions

We defined a pet owner as a participant who, at the time of participation, shares the same residence as at least one of the following animals: dogs, cats, small mammals (rabbits, guinea pigs, hamsters, gerbils, mice, chinchillas) or birds, with the exception of stray animals. We also specifically defined *past* pet ownership as previously sharing the same residence as one of the aforementioned animals prior to participation, excluding current pets. These animals were chosen based on their local prevalence and existing literature reports of the relationship of ownership of these pets on human physical activity, mental health and atopic conditions^4,10,16,22,26,27^. All other participants were considered non-pet owners.

### Survey instruments

We surveyed participants on their sociodemographic profile, past medical history, pet ownership and attachment, physical activity levels, general and mental health, and atopic conditions. Past medical history focused on three common chronic conditions (hypertension, diabetes mellitus, hyperlipidemia) and three psychiatric conditions (depression, anxiety disorders, schizophrenia). Participants could decline to provide information for the mental health conditions. The full questionnaire can be found under Supplementary Material.

#### Relationship between pet owners and pets

We formulated questions about the number and types of pets in each household to accurately determine the nature and temporal sequence of past and present pet ownership. As members in each household may perform different roles in pet ownership, we attempted to delineate such nuances by asking participants if they considered themselves to be the owner and/or the main caregiver for their pet.

The frequency of involvement in routine pet care (feeding, healthcare, activities, hygiene)^28,29^ was assessed on a 5-point Likert scale (from 1 for “Never” to 5 for “All the time”), with a score of 3 or more representing higher involvement in pet care.

We assessed the attachment of pet owners to their pets through 8 questions in which participants rated how much they agreed with each statement on a 5-point Likert scale. The questions were adapted for brevity and comprehension from the Pet Attachment Questionnaire^30^ and the Pet Attachment Survey of the Center for the Study of Human–Animal Relationships and Environments (CENSHARE)^31^. We derived an overall pet attachment score by reverse-scoring of negatively-worded questions and averaging all responses; a score greater than 4 out of 5 represented a close pet-pet owner relationship.

#### Physical activity levels

The Singapore Health Promotion Board (HPB) National Physical Activity Guidelines^32^ defines and classifies physical activity into mild-, moderate- and vigorous-intensity. Mild-intensity physical activity allows one to hold a conversation and even sing during the activity. Moderate-intensity physical activity causes a slight increase in breathing and heart rate, and allows one to hold a conversation but not sing during the activity. Vigorous-intensity physical activity causes a significant increase in breathing and heart rate, and does not allow one to hold a short conversation during the activity. Since physical activity recommendations set out by the health authorities in Singapore are communicated to the general public based on these definitions of physical activity intensity levels, we asked participants to report the average duration per week, in minutes^33^, they engaged in mild-, moderate- and vigorous-intensity aerobic physical activity.

#### 36-item short form survey (SF-36)

The SF-36 is an internationally-used generic health-related quality of life questionnaire with 8 subscales (range 0–100) that can be further categorised into the general and mental health domains. An overall higher score reflects better perceived health. We elected to adapt the publicly available RAND 36-item Health Survey (Version 1.0)^34^ to assess the general and mental health of the participants. Questions pertaining to physical functioning, role limitation due to physical health, and pain scales were excluded as they do not meet the objectives of the study.

#### Atopic conditions

The atopic conditions of interest in this study were asthma, allergic rhinitis and conjunctivitis, and eczema. We adapted the questionnaires from the International Study of Asthma and Allergies in Childhood (ISAAC)^35^ and the The European Community Respiratory Health Survey II (ECRHS II)^36^, and assessed participants regarding the presence and severity of symptoms of these atopic conditions. Participants also reported if a formal diagnosis of any of these conditions were previously made.

To assess the correlation of atopic symptoms with pet *acquisition* and *cessation* of pet ownership, both current and past pet owners with formally-diagnosed atopy were asked to compare their symptom severity (i) while owning *vs* before ever owning a pet, and (ii) when they no longer owned a pet (e.g., after their pet had passed away) *vs* during the period of ownership. These were evaluated on a 5-point Likert scale, with 3 points representing “no change”, and values less than or more than 3 points representing a deterioration or an improvement in symptoms respectively.

### Data pre-processing and cleaning

To ensure the veracity of data entries, we performed manual checking as well as statistical tests for extreme outliers (i.e., Cook’s distance). This enabled identification of likely-erroneous records (e.g., participants who reported > 1000 min of moderate physical activity per week) which were excluded from analyses. Missing data was minimal because the majority of questions in our survey was mandatory, hence analyses could be done on a complete-case basis without need for imputation.

### Statistical analysis

To minimise confounding and selection biases and to facilitate causal inference, we performed comparative analyses of outcomes within a propensity score-matched (PSM) subset of all respondents.

We estimated propensity scores using logistic regression modelling of baseline demographics and other relevant covariates which could predict pet ownership. Several models were developed and compared based on discrimination, calibration and Akaike information criterion^37,38^. The final model consisted of age, race, marriage status, housing type, gender, past pet ownership, and an interaction term between marriage and housing (Supplementary Table S1). This model exhibited good discrimination (area under receiver operating characteristics curve = 0.708, bootstrapped bias-corrected 95% CI: 0.672 to 0.743) and calibration (*p* = 0.452 from Hosmer–Lemeshow test with ten deciles) (Supplementary Figure S1). We conducted propensity score-matching using 1:1 nearest neighbour matching without replacement with a caliper distance of 0.25*standard deviations of the linear predictor (i.e. log odds of the propensity score). Covariate distributions between pet owners and non-pet owners were balanced after conditioning on the propensity score (Supplementary Figure S1 and Table 1).

**Table 1:** Study population baseline demographics and characteristics.

	All individuals (*n* = 823)			Propensity score-matched subset (*n* = 566)		
	Pet owners (*n* = 429)	Non-pet owners (*n* = 394)	*p*-value	Pet owners (*n* = 283)	Non-pet owners (*n* = 283)	*p*-value
**Age, y**^**a**^	33.0 (24.0, 44.0)	27.0 (23.0, 44.0)	0.001	31.0 (24.0, 46.0)	30.0 (23.0, 45.0)	0.867
21–30 (%)	43.1	57.1	< 0.001	48.8	50.2	0.775
31–40 (%)	25.6	14.2		19.4	18.0	
41–50 (%)	19.3	11.7		15.5	14.5	
51–64 (%)	11.9	17.0		16.3	17.3	
Gender, male (%)	17.2	26.9	< 0.001	20.1	22.6	0.167
Singaporean citizen (%)	92.3	93.1	0.506	92.6	92.2	1.000
**Race**						
Chinese (%)	79.5	87.3	< 0.001	86.2	87.3	0.942
Malay (%)	11.7	3.8		4.6	4.9	
Indian (%)	3.7	6.1		4.9	4.2	
Others (%)	5.1	2.8		4.2	3.5	
**Married (%)**	38.2	31.5	0.042	36.7	35.3	0.571
**Housing type**						
HDB flat (%)	62.5	62.2	0.010	59.7	61.8	0.911
Condominium/others (%)	22.1	28.4		27.6	26.5	
Landed (%)	15.4	9.4		12.7	11.7	
**Education level, post-secondary or above (%)**	95.6	94.9	0.662	95.4	94.7	0.845
**Employed (%)**	67.1	57.9	0.006	64.3	60.1	0.307
**Household income > $10,000/month (%)**	38.0	40.1	0.536	41.3	38.5	0.512
**Household members**^**b**^	4 (1–10)	4 (1–9)	0.316^**b**^	4 (1–10)	4 (1–9)	0.706^**b**^
**Past pet ownership (%)**	73.9	49.2	< 0.001	64.0	66.4	0.143
**Medical history**						
Hypertension (%)	6.5	5.1	0.375	6.0	4.6	0.571
Diabetes mellitus (%)	4.4	2.0	0.054	3.2	2.8	1.000
Hyperlipidemia (%)	7.5	5.3	0.214	7.8	6.0	0.458
Depression (%)	7.2	6.6	0.724	7.8	6.7	0.755
Anxiety disorders (%)	8.4	8.4	0.993	9.2	8.8	1.000
Schizophrenia (%)	0.0	0.0	NA	0.0	0.0	NA
**Atopic conditions, formal diagnosis**						
Asthma (%)	19.1	14.0	0.047	19.4	15.5	0.254
Allergic rhinitis (%)	16.6	15.5	0.677	15.5	14.8	0.907
Eczema (%)	21.2	21.6	0.899	19.8	23.3	0.337

^a^ Median (25th percentile, 75th percentile).

^b^ Median (range). Poisson count models were used since the number of household members are non-negative integers that arise from a counting process.

Comparisons of the baseline demographics and characteristics between pet owners *versus* non-pet owners were conducted in both the overall cohort (*n* = 823) as well as the propensity score-matched subset (*n* = 566). In the overall cohort, we performed unadjusted comparisons of respondents’ baseline demographics using Mann–Whitney U test, Pearson’s χ^2^ test, and Poisson regression for continuous, categorical, and count variables respectively. Within the propensity score-matched cohort, we used the ‘paired’ equivalents of the aforementioned tests: hence, the Wilcoxon signed-rank test, McNemar’s χ^2^ test, and mixed-effects Poisson models were used for continuous, categorical, and count demographic variables respectively.

Comparisons of physical activity levels and SF-36 outcomes between pet owners versus non-pet owners were performed within the propensity score-matched cohort. Because the matched design induces clustering of standard errors and correlation of responses within matched pairs, we used maximum likelihood mixed-effects linear models to estimate the average treatment effect of pet ownership for continuous outcomes. We performed subgroup analyses by incorporating a full factorial interaction between pet ownership and categorical moderator variables (i.e., the subgroup of interest) into the mixed-effects models, and calculating post-estimation subgroup-specific marginal effects. All subgroup analyses used observations from only the propensity score-matched subset of respondents. These covariates were chosen for investigation based on associations reported between the outcome measures and these variables (a full list of supporting references are provided in Supplementary Table S2). It should be noted that comparisons in the subgroup analyses are made between the relevant subset of pet owners and a comparator subset comprising their corresponding matched non-pet owners.

We also examined the impact of pet ownership on symptom severity in respondents with formally-diagnosed atopic conditions (asthma, allergic rhinitis, or eczema). As explained previously, self-reported changes in severity of atopic symptoms upon current pet ownership and loss of past pet ownership (i.e., when the pet was no longer in the household) were rated on a 5-point Likert scale in which 3 points represents “no change”, and values less than or more than 3 points represent deterioration or improvement in symptoms respectively. Therefore, we used a single-sample *t*-test under the null hypothesis that there is no change in symptom severity (i.e., 3 points).

Statistical analyses were performed in Stata version 16.0 (StataCorp, https://www.stata.com/products/), and two-sided nominal *p* < 0.05 were considered to indicate statistical significance. In view of the subgroup analyses for each outcome measure, it should be noted that under the Bonferroni correction procedure for multiplicity, the adjusted significance threshold should be ~ 0.002 (under the most conservative assumptions) in our study in order to preserve the family-wise error rate at 0.05. However, as potential controversy may arise concerning the (i) optimal multiplicity-correction procedure to use, (ii) the increasing advocacy for researchers to focus on effect sizes and their precision (or 95% confidence intervals), and (iii) advocacy for researchers to consider their results under a Bayesian perspective, we encourage readers to focus on the (a) effect sizes reported herein and (b) the plausibility of the explanations for the results we proffer in the subsequent discussion, which could update the posterior probability of a hypothesis being true. Finally, we also opted to (c) report nominal *p* values to allow informed readers the flexibility to re-interpret the significance of our nominal results with their preferred optimal method of multiplicity-correction. We recommend that for the purpose of frequentist-driven interpretation, readers may regard *p* values < 0.002 as statistically significant, while *p* values from 0.002 to 0.05 should be interpreted with greater caution.

## Results

### Baseline demographics

Table 1 summarises the baseline demographics of the unmatched and matched cohorts of respondents. Of 823 respondents in the overall unmatched cohort, 429 (52.1%) were pet owners and 384 (47.9%) were non-pet owners. We detected significant imbalances when comparing unmatched data of pet owners and non-pet owners in terms of age, gender, race, marital status, housing type, employment and past pet ownership (Table 1). The median age of pet owners (33.0 years [IQR 24.0–44.0]) was higher than non-pet owners (27.0 years [IQR 23.0–44.0]) (*p* = 0.001). More pet owners were married (164/429 [38.2%] *vs* 124/394 [31.5%], *p* = 0.042), employed (288/429 [67.1%] *vs* 228/394 [57.9%], *p* = 0.006) and had previous pets (317/429 [73.9%] *vs* 193/394 [49.2%], *p* < 0.001).

Following 1:1 propensity score-matching, there were 283 participants in each arm for a total of 566 respondents in the matched set, and both arms were well-balanced in all demographic variables (Table 1).

### Physical activity levels

Pet owners did not report any difference in duration of mild- (*β* = 10.8, 95% CI: − 3.90 to 25.6; *p* = 0.150), moderate- (*β* =  − 0.6, 95% CI: − 7.30 to 6.10; *p* = 0.866), or vigorous-intensity (*β* =  − 3.0, 95% CI: − 8.10 to 2.10; *p* = 0.251) physical activity compared to non-pet owners.

**Figure 1:**
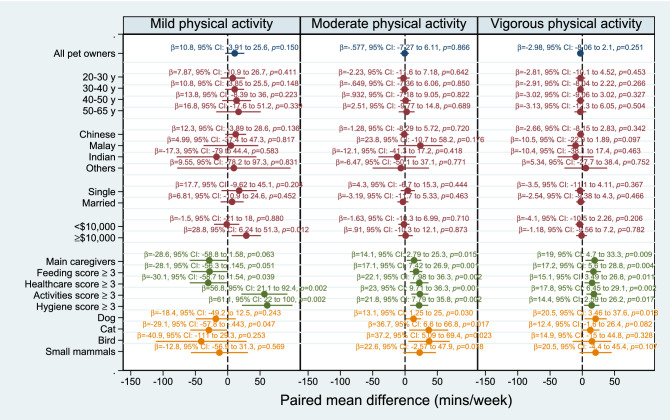
Propensity score-matched comparison of weekly physical activity levels between pet owners *vs* non-pet owners in the full matched set as well as selected subgroups. Subgroup-specific effects were computed as marginal contrasts by specifying a full factorial interaction between pet ownership and the relevant covariate. Subgroup analyses for gender, housing type, education level, employment status and pet attachment score did not yield statistically significant results (i.e., all *p*-values obtained were greater than 0.05) and are not shown here. The full results are presented in Supplementary Figure S2.

Full results for subgroup analyses are shown in Fig. 1 and Supplementary Figure S2. For mild-intensity physical activity levels, pet owners with a caregiver (healthcare) score ≥ 3 had lower activity levels of *β* =  − 30.1 (95% CI: − 58.7 to − 1.54; *p* = 0.039) minutes/week than their matched counterparts in the non-pet owner cohort, as did cat owners (*β* =  − 29.1, 95% CI: − 57.8 to − 0.443; *p* = 0.047). Pet owners with a caregiver (activities) score ≥ 3 (*β* = 56.8, 95% CI: 21.1 to 92.4; *p* = 0.002); caregiver (hygiene) score ≥ 3 (*β* = 61.1, 95% CI: 22.0 to 100; *p* = 0.002) and monthly household income of greater than $10,000 (*β* = 28.8, 95% CI: 6.24 to 51.3; *p* = 0.012) exhibited higher mild-intensity physical activity levels than non-pet owners.

Main caregivers (*β* = 14.1, 95% CI: 2.79 to 25.3; *p* = 0.015), dog owners (*β* = 13.1, 95% CI: 1.30 to 25.0; *p* = 0.030), cat owners (*β* = 36.7, 95% CI: 6.60 to 66.8; *p* = 0.017) and bird owners (*β* = 37.2, 95% CI: 5.09 to 69.4; *p* = 0.023) demonstrated higher moderate-intensity physical activity levels than non-pet owners. Pet owners in all caregiver subgroups (feeding score ≥ 3 [*β* = 17.1, 95% CI: 7.42 to 26.9; *p* = 0.001]; healthcare score ≥ 3 [*β* = 22.1, 95% CI: 7.98 to 36.3; *p* = 0.002]; activities score ≥ 3 [*β* = 23.0, 95% CI: 9.70 to 36.3; *p* = 0.001]; hygiene score ≥ 3 [*β* = 21.8, 95% CI: 7.79 to 35.8; *p* = 0.002]) also exhibited higher moderate-intensity physical activity levels than non-pet owners.

The following subgroups of pet owners demonstrated higher levels of vigorous-intensity physical activity than non-pet owners: main caregivers (*β* = 19.0, 95% CI: 4.70 to 33.3; *p* = 0.009); caregiver (feeding) score ≥ 3 (*β* = 17.2, 95% CI: 5.60 to 28.8; *p* = 0.004); caregiver (healthcare) score ≥ 3 (*β* = 15.1, 95% CI: 3.49 to 26.8; p = 0.011); caregiver (activities) score ≥ 3 (*β* = 17.8, 95% CI: 6.50 to 29.1; *p* = 0.002); caregiver (hygiene) score ≥ 3 (*β* = 14.4, 95% CI: 2.60 to 26.2; *p* = 0.017); and dog ownership (*β* = 20.5, 95% CI: 3.50 to 37.6; *p* = 0.018).

### Subjective well-being (SF-36 subscales)

**Figure 2:**
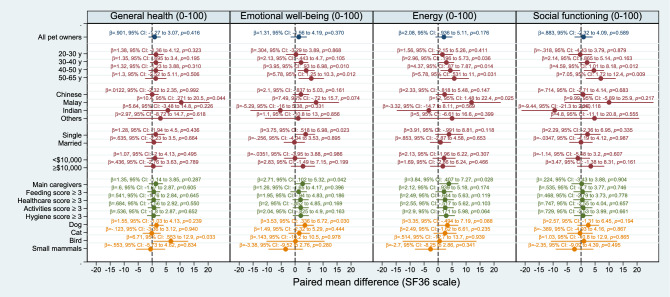
Propensity score-matched comparison of SF-36 subjective domain scores between pet owners *vs* non-pet owners in the full matched set as well as selected subgroups. Subgroup-specific effects were computed as marginal contrasts by specifying a full factorial interaction between pet ownership and the relevant covariate. Subgroup analyses for gender, housing type, education level, employment status and pet attachment score did not yield statistically significant results (i.e., all *p*-values obtained were greater than 0.05) and are not shown here. The full results are presented in Supplementary Figure S3.

There were no differences in emotional well-being, social functioning, general health, and energy levels between pet owners and non-pet owners in the overall matched cohort (Fig. 2).

We performed further subgroup analyses to investigate whether pet owners belonging to specific demographic strata may experience differential effects (Fig. 2 and Supplementary Figure S3). Malay pet owners had better general health scores compared to non-pet owners (*β* = 10.4, 95% CI: 0.270 to 20.5; *p* = 0.044). Bird owners also demonstrated higher general health scores (*β* = 6.7, 95% CI: 0.550 to 12.9; *p* = 0.033) than non-pet owners.

Pet owners aged 41–50 years (*β* = 4.0, 95% CI: 0.930 to 6.98; *p* = 0.010) and 51–64 years (*β* = 5.8, 95% CI: 1.25 to 10.3; *p* = 0.012) exhibited higher emotional well-being scores than non-pet owners. The following pet owner subgroups also demonstrated higher emotional well-being scores compared to their matched counterparts in the non-pet owner cohort: single marital status (*β* = 3.8, 95% CI: 0.529 to 6.98; *p* = 0.023); main caregiver (*β* = 2.7, 95% CI: 0.100 to 5.32; *p* = 0.042); and dog owners (*β* = 3.5, 95% CI: 0.340 to 6.72; *p* = 0.030).

With regards to energy levels, the following subgroups of pet owners demonstrated higher scores than non-pet owners: age 31–40 years (*β* = 3.0, 95% CI: 0.200 to 5.73; *p* = 0.036); age 41–50 years (*β* = 4.4, 95% CI: 0.670 to 7.87; *p* = 0.014); age 51–64 years (*β* = 5.8, 95% CI: 0.530 to 11.0; *p* = 0.031); Malay race (*β* = 12.0, 95% CI: 1.48 to 22.4; *p* = 0.025); and main caregivers (*β* = 3.8, 95% CI: 0.410 to 7.27; *p* = 0.028).

Similarly, higher social functioning scores were seen in pet owners aged 41–50 years (*β* = 4.6, 95% CI: 1.01 to 8.18; *p* = 0.012) and 51–60 years (*β* = 7.05, 95% CI: 1.72 to 12.4; *p* = 0.009) as compared to non-pet owners.

### Atopy

There were no significant differences in prevalences of formally-diagnosed allergic rhinitis and eczema between the unmatched cohorts of pet and non-pet owners. However, formally-diagnosed asthma was more prevalent amongst pet owners (82/429 [19.1%]) than non-pet owners (55/394 [14.0%]) in the unmatched overall cohort (*p* = 0.047), but this imbalance was ablated after propensity score-matching (Table 1).

**Figure 3:**
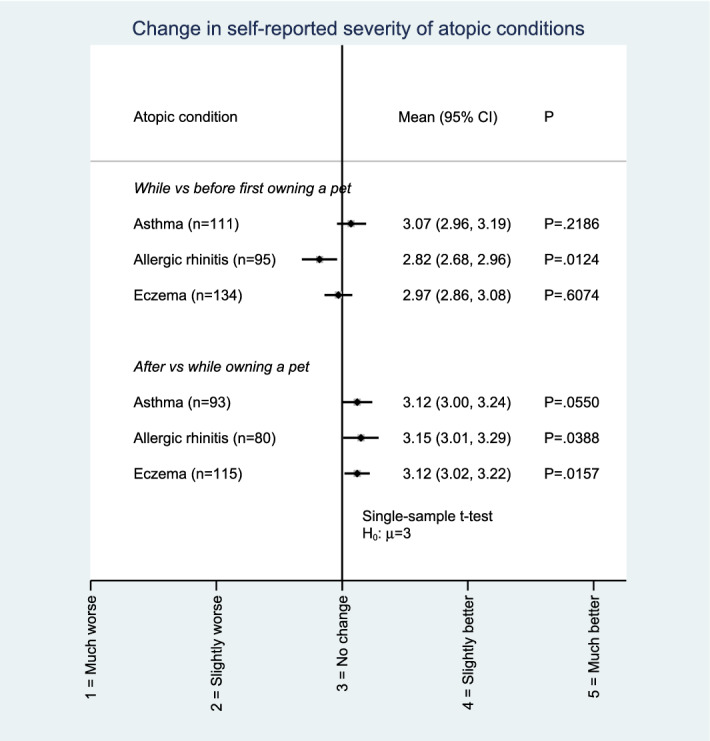
Changes in self-reported symptom severity among subgroups of participants with previously-diagnosed atopic diseases. Comparisons were made in regards to symptom severity after *vs* before they owned a pet, and when they no longer owned a pet (e.g., after their pet passed away) *vs* during the time they owned a pet. A single-sample *t*-test was used to compare against a null of 3 points, which denotes "no change" in symptom severity on the Likert scale in the study questionnaire.

We performed a further analysis—in which current and past pet owners with formally-diagnosed atopic conditions served as their own controls—to determine if there were temporal differences in self-reported symptom severity over the course of pet ownership: ‘While owning a pet’ *vs* ‘Before first owning a pet’ and ‘After the pet is no longer around’ *vs* ‘While owning a pet’ (Fig. 3). In the first comparison, current pet owners reported worsening of their current allergic rhinitis symptoms (single-sample mean: 2.82, 95% CI: 2.68 to 2.96; *p* = 0.012 [compared to the null hypothesis of *μ* = 3 points]) after acquiring their current pet(s). In the second comparison, past pet owners reported improvements in allergic rhinitis (mean: 3.15, 95% CI: 3.01 to 3.29; *p* = 0.039) and eczema symptoms (mean: 3.12, 95% CI: 3.02 to 3.22; *p* = 0.016) in the past after cessation of past pet ownership. No differences in asthma symptom severity in either of the comparisons were observed.

### Continuous-by-categorical interactions and effects modifiers

To yield greater insight for future studies, we sought to elucidate the existence of continuous-by-categorical effect modifiers and statistical interactions.

**Figure 4:**
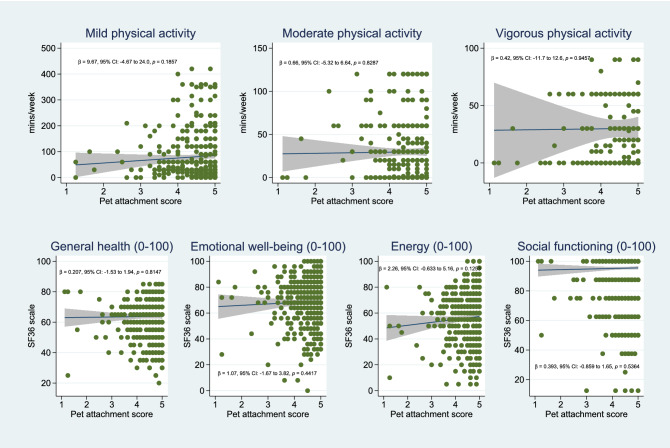
Analysis of effect modification by overall pet attachment score, which was calculated as the average score of *n* = 8 questions adapted from the Pet Attachment Questionnaire by Zilcha-Mano et al. (2011) ^30^. Note that only pet owners were able to provide responses to questions on pet attachment, and hence only they were included in these analyses. Similar conclusions were obtained in sensitivity analyses using Poisson count and zero-inflated Poisson models for duration of physical activity per week (data not shown).

We found that pet attachment scores did not modify the association between pet ownership and physical activity levels or SF-36 subscales (Fig. 4).

**Figure 5:**
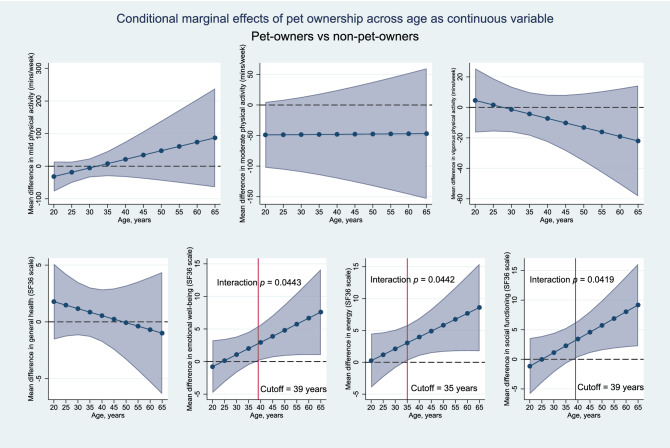
Average marginal effects (95% CI) of pet ownership are plotted across age, when age is analysed as a continuous variable and a full factorial interaction between age and pet ownership is specified. *p* values for interaction terms are shown if statistically significant. These analyses suggest that the benefits of pet ownership on emotional well-being, energy, and social functioning accrue with increasing age, with statistically significant benefits for participants above the age of 39, 35 and 39 years old respectively.

We also examined whether age constitutes a moderator variable by incorporating a full factorial interaction between age (as a continuous variable) and pet ownership, and computing the average marginal effects of pet ownership on physical activity levels and SF-36 subscales over age (Fig. 5). These analyses suggest that older pet owners had statistically greater emotional well-being, energy and social functioning scores than matched non-pet owners, specifically when they were above the ages of 39 (interaction *p* = 0.043), 35 (interaction *p* = 0.044) and 39 (interaction *p* = 0.042) years old respectively. These differences further increased with increasing age. However, there was no statistically-significant interaction between pet ownership and age when analysing physical activity levels (mild, moderate and vigorous) and general health.

## Discussion

In this study, pet ownership was shown to have several significant associations with physical activity levels, mental health and atopic conditions among adults aged 21–64 years. However, some of these associations are restricted to certain subgroups in this study, illustrating their heterogeneity and complex interactions with sociodemographic factors, pet types and caregiver role involvements.

There was no significant association of current pet ownership with physical activity levels in the overall matched cohort. Our findings agree with that of Taniguchi et al., who found no significant association of moderate-to-vigorous physical activity with dog or cat ownership in their cohort^4^. However, our subgroup analyses revealed that main pet caregivers had higher moderate- and vigorous-intensity physical activity levels than non-pet owners, likely attributable to caregiving- and pet-related physical activities (e.g., dog walking), although the interpretation should be made in context of multiple testing. Furthermore, our results add nuance to current research by recognising that pet ownership is a spectrum—from passive sharing of residence to active engagement with the pet. In our study, we observed higher moderate- and vigorous-intensity physical activity levels in current dog owners compared to non-pet owner counterparts, a finding also seen in previous studies^39,40^ but should also be interpreted with caution as the *p* values are larger than 0.002. This was not observed for other types of pets such as cats, birds, and other small mammals. The unique association of dog ownership with higher levels of moderate- and vigorous-intensity physical activity may be due to closer companionship^41^, dogs being inherently more physically active^42^, as well as specific physical activities (including hiking, swimming and agility training) that pet owners are more likely to engage in with dogs as compared to other pet types. Taken together, our findings indicate that the association of current pet ownership with physical activity levels may be influenced by the type of pet and the degree of engagement with pets.

Pet ownership has been described to positively impact mental health through a range of mechanisms including reducing stress^43,44^, promoting social interaction^45,46^, classical conditioning of relaxation response, providing emotional support and tactile interactions^47^, creating a non-threatening atmosphere^12^, catalysing the development of social support networks^48^, as well as boosting self-esteem^13^. In our overall propensity score-matched cohort, we did not detect a significant association of pet ownership with any of the four SF-36 subscales studied. However, our subgroup analyses revealed significant treatment effect heterogeneity across demographics subgroups of age, marital status, pet type and caregiver role, although none achieved a *p* value less than 0.002. We found age to be a moderator variable, with associations between pet ownership and mental health only becoming significant above the ages of 39, 35 and 39 years for emotional well-being, energy and social functioning respectively. In a similar vein, Nagasawa & Ohta showed that childhood dog ownership was associated with increased sociality in old age, suggesting possible cumulative effects of pet ownership over a person’s lifetime, with greater benefits at an older age^49^. Our study also found that current pet owners who considered themselves the main pet caregivers as well as those who were at least sometimes involved in certain caregiving activities reported higher emotional well-being and energy scores compared to their matched non-pet owner counterparts, which underscores the notion that meaningful pet engagement is required to derive the mental health benefits of owning a pet. Furthermore, we found that current dog owners scored significantly higher in emotional well-being compared to non-pet owners, while owners of other animal types did not. This may be explained by the close physical and emotional relationship dogs provide^50^, which has led to the wide use of dogs in animal-assisted therapies and interventions^51–53^. Intriguingly, our study also found that non-married pet owners reported better emotional well-being compared to non-pet owners, whereas this was not seen in their married counterparts. We posit that the different experiences of married and unmarried pet owners can be explained by the notion that marriage provides emotional support between spouses which tempers the amount of emotional fulfilment and satisfaction that pet companionship can further afford. Furthermore, being unmarried for a longer duration is associated with higher levels of depression^54,55^, hence the potential benefits of owning a pet on emotional well-being may be more apparent among non-married respondents.

In those with a formal diagnosis of atopic conditions, we found that self-reported symptoms of allergic rhinitis worsened with pet acquisition while those of allergic rhinitis and eczema subsequently improved when pet ownership ceased. Pets may serve as allergen sources^22–24,56^, with a study showing that fur-bearing animals exacerbated symptoms of pre-existing perennial allergic rhinitis^57^. Conversely, other studies have described that pet ownership may be associated with a reduced risk of developing atopic diseases^58^ as a result of allergen desensitisation^16–18^. However, our findings should be interpreted with caution because of the following limitations. Firstly, our survey did not probe the temporal relationship between onset of atopic symptoms and pet ownership in detail. Secondly, our cross-sectional study design is inefficient for decoupling any worsening or improvement of atopic symptoms from the natural disease course, which is an important confounder since atopic conditions are generally thought to remit over time. Thirdly, this analysis is particularly subject to recall bias, as participants are expected to recollect their symptoms over a long time interval (e.g., from childhood). Therefore, this research question would be better answered using a carefully-designed prospective longitudinal study with objective clinical and/or biochemical evidence (e.g., serum antibody measurements).

Strengths of the current study include its quasi-experimental study design—propensity score-matching—which supports causal inference while mitigating both selection and confounding biases. The advantages of propensity score-matching over traditional covariance adjustment for estimating treatment effects include but are not limited to: (i) avoiding the need to specify a functional relationship between confounders and the outcomes of interest, which could otherwise be susceptible to model misspecification; (ii) permitting the estimation of causal estimates of the average treatment effect as opposed to merely demonstrating associations, because propensity score-matching adheres to the ‘counterfactual’ or ‘potential outcomes’ framework of causality; (iii) enabling dimensionality reduction because confounders are reduced to a single scalar variable (i.e., the propensity score). Furthermore, in this study, treatment effect sizes for key subgroups were calculated by modelling the treatment-by-covariate interaction (i.e., pet ownership##covariate), which makes use of all observations more efficiently and improves the statistical power of the analysis (as compared to simple stratified analysis wherein a subset of observations are dropped during computation of subgroup-specific treatment effects). Other highlights of our study include characterising the type and degree of caregiver involvement of pet owners as well as quantifying pet attachment levels, which fills a lacuna because most published literature in this avenue of research have simply dichotomised respondents into pet owners and non-pet owners. This detailed characterisation and sub-typing of pet owners allowed us to discover the differential effects that pet ownership may have on physical activity and mental health across distinct caregiver roles.

Limitations of our study include the fact that our study population comprises a majority of young respondents, with 49.8% of all respondents in the age range of 21–30 years. In comparison, 13.3% of the Singapore population is aged 20–29 years^59^. This may be a consequence of the survey methodology using an online questionnaire, which limits participants to those with digital devices and internet access. Furthermore, owing to the need for informed consent and cognitive screening in potential elderly respondents, as well as the inability to effectively administer the Mini-Cog screening tool^60^ online, we were advised by the Departmental Ethics Review Committee to exclude elderly persons aged 65 years and above from the survey. The general health of our study population is high since it mostly comprised of young healthy individuals, and therefore it remains to be seen how the influence of pet ownership may be augmented by the presence of common chronic medical conditions, the prevalence of which increases with age^33^. Despite subgroup analyses suggesting that positive health benefits may accrue to older participants, the exclusion of these individuals could have attenuated or “diluted” the magnitude of the potential benefits of pet ownership on the older population when we carried out the analysis for the overall study population. In addition, a small sample size was yielded for this study, reducing the statistical power of our analyses. Nevertheless, the adoption of a propensity score-matched design reduces selection bias by emulating randomisation, which may therefore attenuate some of the selection bias associated with the use of voluntary online surveys as means of selecting participants. Another limitation of our work is the issue of multiple testing, which could be associated with an elevated type 1 error rate. In our study, we did not attempt to adjust the type 1 error rate for the multiplicity of hypotheses tested, owing to the exploratory nature of the research, and instead acknowledge that the *p* values reported are nominal and also recommend that readers focus on effect sizes and their confidence intervals. As explained previously, *p* values < 0.002 may be regarded as statistically significant under the conservative Bonferroni correction procedure, while *p* values from 0.002 to 0.05 should be interpreted with greater caution. The explanations we have provided in the discussion may also lend support to the truth of our findings.

Our study affords new insights into the health-related benefits of pet ownership, which can help to guide further studies and inform future public health interventions regarding pets. Firstly, increasing pet exposure in the older adult population may improve their mental health and physical activity levels. However, since the elderly also have significantly more health conditions or financial constraints which may hinder them from caring for their pets^61^, one solution is to organise dog walking opportunities for elderly people, hence allowing the elderly to reap the benefits of pet ownership without subjecting them to the financial costs and burden of caring for a pet. Initiated by local community organisations, charities and animal shelters, these programmes can also allow animals in these shelters to socialise frequently and increase their chances of adoption. Secondly, our findings support the promotion of pet ownership among younger adults. Given the rising prevalence of mental health conditions among younger adults, they may be poised to benefit from interventions to improve their mental well-being. Pet ownership can be a novel means of improving emotional well-being amongst the youth. It is well known that reducing stress and developing encouraging support networks are important in the prevention and management of mental health conditions^62,63^. Pet ownership can achieve this two-fold aim by (i) helping to reduce anxiety and stress levels^44,64^, which are major triggers for mental health conditions, and (ii) promoting social interaction and developing new social supports^65^. However, the realities of owning a pet at a young age may be daunting and unfeasible to most. An alternative would be to increase pet exposure among non-pet owners by promoting pet-petting cafes and providing volunteering opportunities at animal organisations. Introducing therapy dog workshops in schools can also help students to manage stress and overcome anxiety^13,66^. Thirdly, our findings also support pet-assisted therapies for hospital inpatients, palliative care patients, psychiatric patients and nursing home residents. There is a growing body of supportive evidence for animal-assisted therapy^10,67,68^ as a form of social support^69^ to improve mental health^70^, which has clinical implications in cognitive function^71,72^, mental disabilities and developmental disorders^10,73^. Pet therapy has also been shown to significantly reduce self-reported stress and anxiety levels^43^. Hence, pet therapy has potential to complement conventional treatments in alleviating pain and anxiety, and improve quality of life. Lastly, further research on atopy to investigate the immunological mechanisms behind the positive or adverse effects of pet exposure on atopic conditions will be useful to support or refute the findings of this study and other studies with similar results^22,24^. Some of the processes implicated in allergen desensitisation include a modified T-helper (Th)2-cell response with induction of IgG4 expression while suppressing IgE^74^ and boosted interleukin (IL)-10 responses via increased endotoxin exposure^75^. A prospective longitudinal study can be further undertaken to determine causality between pet ownership and atopic conditions, eliminating recall bias and controlling for the natural disease progression. The results of these studies can inform healthcare professionals to better make key recommendations to pet owners with atopic conditions with regards to reduction of allergen exposure^76^.

## Conclusion

To our knowledge, this is the first study examining the public health impact of pet ownership in Southeast Asia, offering directions for future research on pet ownership and its associated health effects. Although there were no differences in physical activity levels and mental health scores between all pet owners and non-pet owners in the propensity score-matched set, subgroup analyses revealed that main pet caregivers had higher physical activity levels and higher scores in the various mental health domains. With respect to mental health, an interaction was found between age and pet ownership, whereby mental health was positively associated with pet ownership, accruing with the increasing age of pet owners. Additionally, higher levels of emotional well-being were experienced by non-married pet owners compared to their non-married non-pet owner counterparts. Finally, it was observed that self-reported severity of allergic rhinitis symptoms increased during the period of current pet ownership, with subsequent improvements in symptoms of allergic rhinitis and eczema after cessation of past pet ownership. Our findings add contextual nuance to prevailing research, indicating that these specific demographic subgroups may potentially experience both beneficial and adverse outcomes from pet ownership.

## Supplementary information

Supplementary Information

## Data Availability

The authors will publish the dataset in a publicly-available online repository 1 year after the publication of this manuscript.
