# Publisher Correction: Association between pet ownership and physical activity levels, atopic conditions, and mental health in Singapore: a propensity score-matched analysis

**DOI:** 10.1038/s41598-021-85732-2

**Published:** 2021-03-11

**Authors:** Ying Xian Goh, Joel Shi Quan Tan, Nicholas L. Syn, Beverley Shu Wen Tan, Jia Ying Low, Yi Han Foo, Waikit Fung, Brandon Yi Da Hoong, Junxiong Pang, Phase IV CHP 2020 Group 8, Qi Xuan Lim, Jieying Wee, Terence Yan Ming Ng, Hsin Han Elisha Chow, Yu Ling Ng, Jiamin Charmaine Chong, Charmaine Yan Yeo, Lorraine Hui En Tan, Abigail E Xuan Sim, Ahmad bin Hanifah Marican Abdurrahman, Carissa-Jill Yinn Soon, Ian Jun Yan Wee, Julia Yu Xin Ng, Xin Chen Lim, Lloyd Jee Hean Ng, Mervin Nathan Han Hui Lim, Wei Ren Ong, Wen Tao Daniel Ong, Ryan Gabriel Tan, S. Hema Viganeshwari, Santhosh S/O Sasidaran Pillai, Shawn Soon Han Chan, Siti Humaira Bte Mohd Kamil, Isabel Soh, Mengyue Su, Yu Xiang Tan, Valerie Tian Wei Chew, Lily Wei Yun Yang, Mun Yike Fiona Yee

**Affiliations:** 1 Yong Loo Lin School of Medicine, National University of Singapore and National University Health System, Singapore, Singapore; 2 Saw Swee Hock School of Public Health, National University of Singapore and National University Health System, 12 Science Drive 2, Level 10, Singapore, 117549 Singapore; 3 Centre for Infectious Disease Epidemiology and Research, National University of Singapore, Singapore, Singapore

Correction to: *Scientific Reports* 10.1038/s41598-020-76739-2, published online 16 November 2020

In the original version of this Article, the author Brandon Yi Da Hoong was indexed incorrectly. This error has now been corrected.

