# Supplementary material for: Association between pet ownership and physical activity levels, atopic conditions, and mental health in Singapore: a propensity score-matched analysis
Source: Sci Rep. 2020 Nov 16;10:19898. doi: 10.1038/s41598-020-76739-2 (PMC7670461; doi:10.1038/s41598-020-76739-2)
Supplement: Supplementary file 1 — Supplementary Information [file 41598_2020_76739_MOESM1_ESM.pdf]

Supplementary Material

Association between Pet Ownership and Physical Activity Levels, Atopic Conditions, and Mental Health in Singapore: A Propensity Score-Matched Analysis

Ying Xian Goh[1], Joel Shi Quan Tan[1], Nicholas L. Syn[1], Beverley Shu Wen Tan[1], Jia Ying Low[1], Yi Han Foo[1], Waikit Fung[1], Brandon Yi Da Hoong[1], Phase IV CHP 2020 Group 8[1], Junxiong Pang[2,3,*]

[1]Yong Loo Lin School of Medicine, National University of Singapore and National University Health System, Singapore
[2]Saw Swee Hock School of Public Health, National University of Singapore and National University Health System, Singapore
[3]Centre for Infectious Disease Epidemiology and Research, National University of Singapore, Singapore

*Correspondence: Junxiong Pang, MSc, PhD; Saw Swee Hock School of Public Health, National University of Singapore, 12 Science Drive 2, Level 10, Singapore 117549, Republic of Singapore; E-mail: ephpjv@nus.edu.sg

Eligibility Criteria (in English)

Inclusion Criteria:

- Adults from 21 to 64 years of age residing in Singapore for the last 6 months.
- Participants may be clients of licensed veterinary clinics, who are pet-owners/caregivers of the above-mentioned pets.

Exclusion Criteria:

- Legal minors under the age of 21 years.
- Adults aged 65 years and above.
- People who own therapy or guide dogs.
- People with physical disabilities, defined as requiring assistance in at least 1 activity of daily living (ADLs): dressing, eating, ambulating, transferring, toileting, maintaining hygiene.

Questionnaire (in English)

Screening questions before questionnaire

1.	I am between 21 and 64 years of age (inclusive) and am willing to participate in this study.	☐ Click here to acknowledge
2.	I have not previously participated in this study.	☐ Click here to acknowledge
3.	To the best of my knowledge, no one else within my household has previously participated in this study.	☐ Click here to acknowledge

Main questionnaire

4.	What is your age? How old are you this year?	____________________
5.	What is your gender?	☐ Male ☐ Female
6.	What is your nationality?	☐ Singaporean → **Go to 9**<br>☐ Permanent Resident (PR) → **Go to 8**<br>☐ Others → **Go to 7**
7.	Please specify:	____________________
8.	For how long have you been living in Singapore?	____________________
9.	What is your race?	☐ Chinese → **Go to 11**<br>☐ Malay → **Go to 11**<br>☐ Indian → **Go to 11**<br>☐ Others → **Go to 10**
10.	Please specify:	____________________
11.	What is your current marital status?	☐ Single ☐ Married<br>☐ Divorced/separated ☐ Widowed
12.	What is your housing type?	☐ HDB Rental Flat<br>☐ HDB 1-room Flat<br>☐ HDB 2-room Flat<br>☐ HDB 3-room Flat<br>☐ HDB 4-room Flat<br>☐ HDB 5-room Flat<br>☐ HDB Executive Flat<br>☐ Condominium and Other Apartments<br>☐ Landed Property

13.	Postal code<br>*Please select the first 2 digits of your postal codes from the drop down list*	☐ 01, 02, 03, 04, 05, 06 - Raffles Place, Cecil, Marina, People's Park<br>☐ 07, 08 - Anson, Tanjong Pagar<br>☐ 09, 10 - Telok Blangah, Harbourfront<br>☐ 11, 12, 13 - Pasir Panjang, Hong Leong Garden, Clementi New Town<br>☐ 14, 15, 16 - Queenstown, Tiong Bahru<br>☐ 17 - High Street, Beach Road (part)<br>☐ 18, 19 - Middle Road, Golden Mile<br>☐ 20, 21 - Little India<br>☐ 22, 23 - Orchard, Cairnhill, River Valley<br>☐ 24, 25, 26, 27 - Ardmore, Bukit Timah, Holland Road, Tanglin<br>☐ 28, 29, 30 - Watten Estate, Novena, Thomson<br>☐ 31, 32, 33 - Balestier, Toa Payoh, Serangoon<br>☐ 34, 35, 36, 37 - Macpherson, Braddell<br>☐ 38, 39 40, 41 - Geylang, Eunos<br>☐ 42, 43, 44, 45 - Katong, Joo Chiat, Amber Road<br>☐ 46, 47, 48 - Bedok, Upper East Coast, Eastwood, Kew Drive<br>☐ 49, 50, 81 - Loyang, Changi<br>☐ 51, 52 - Tampines, Pasir Ris<br>☐ 53, 54, 55, 82 - Serangoon Garden, Hougang, Punggol<br>☐ 56, 57 - Bishan, Ang Mo Kio<br>☐ 58, 59 - Upper Bukit Timah, Clementi Park, Ulu Pandan<br>☐ 60, 61, 62, 63, 64 - Jurong<br>☐ 65, 66, 67, 68 - Hillview, Dairy Farm, Bukit Panjang, Choa Chu Kang<br>☐ 69, 70, 71 - Lim Chu Kang, Tengah<br>☐ 72, 73 - Kranji, Woodgrove<br>☐ 75, 76 - Yishun, Sembawang<br>☐ 77, 78 - Upper Thomson, Springleaf<br>☐ 79, 80 - Seletar
14.	What is your highest level of education attained?	☐ No Qualification / Pre-primary / Lower primary education<br>☐ Primary<br>☐ Secondary<br>☐ Post-Secondary (Non-Tertiary)<br>☐ Polytechnic Diploma<br>☐ Professional Qualification<br>☐ University (Bachelor's or Equivalent)<br>☐ Post-graduate Diploma / Certificate Qualification / Master's / Doctorate<br>☐ Others

15.	What is your employment status?<br>*Employee refers to a person who works for employers in return for regular wages or salaries.*<br>*Employer refers to a person who employs at least one paid worker in their business or trade.*<br>*Own account worker (i.e. self-employed) refers to a person who operates his/her own business without employing any paid workers in the conduct of his/her business or trade.*<br>*Contributing family worker refers to a person who assists in the operation of family business without receiving regular wages or salaries.*	☐ Employee<br>☐ Domestic Helper<br>☐ Employer<br>☐ Own account worker (i.e. self-employed)<br>☐ Contributing family worker
16.	What is your employment type?	☐ Full-time<br>☐ Part-time<br>☐ Contract worker
17.	What is your gross monthly household income?<br>*This includes the income of everyone living in your household, before tax/CPF contribution. Please provide an estimate even if you are not certain about the exact value.*	☐ Less than $5000<br>☐ $5000 to $9999<br>☐ $10,000 to $14,999<br>☐ $15,000 to $19,999<br>☐ $20,000 and above
18.	How many members are there in your household, including yourself?	____________________

<u>Section Header: Pet ownership</u>

19.	Are there any pets in your household?<br>*This includes any pets which may belong to any members of your household. Pets include: dogs, cats, birds, small mammals (e.g. rabbits, guinea pigs, hamsters, gerbils, mice, chinchillas)*	☐ Yes → **Go to 20**<br>☐ No → **Go to 42**
20.	Are you the owner of the pet?<br>*Pets include: dogs, cats, birds, small mammals (e.g. rabbits, guinea pigs, hamsters, gerbils, mice, chinchillas)*	☐ Yes<br>☐ No
21.	Do you consider yourself the main caregiver of the pet?	☐ Yes<br>☐ No

22. How often are you the main person in charge of the following needs of your pet? [1,2]

23.	Feeding	☐ Never<br>☐ Rarely<br>☐ Sometimes<br>☐ Most of the time<br>☐ All the time
24.	Healthcare	☐ Never<br>☐ Rarely<br>☐ Sometimes<br>☐ Most of the time<br>☐ All the time
25.	Activities: walking, playing	☐ Never<br>☐ Rarely<br>☐ Sometimes<br>☐ Most of the time<br>☐ All the time
26.	Hygiene: grooming, showering	☐ Never<br>☐ Rarely<br>☐ Sometimes<br>☐ Most of the time<br>☐ All the time

27. What type(s) of pets do you currently own / have in your household?
You may select multiple options

☐ Dog
→ **Go to 28-29**

28. How many dogs do you currently own? ____________

29. For how many years have you owned your current dog(s)? ____________
If you own more than 1 dog, please list the number of years you own each dog individually (for example, type "5, 4, 2" if you have owned 3 dogs for 5, 4 and 2 years each).
6 months = 0.5 years

☐ Cat
→ **Go to 30-31**

30. How many cats do you currently own? ____________

31. For how many years have you owned your current cat(s)? ____________
If you own more than 1 cat, please list the number of years you own each cat individually (for example, type "5, 4, 2" if you have owned 3 cats for 5, 4 and 2 years each).
6 months = 0.5 years

☐ Bird
→ **Go to 32-33**

32. How many birds do you currently own? ____________

33. For how many years have you owned your current bird(s)? ____________
If you own more than 1 bird, please list the number of years you own each bird individually (for example, type "5, 4, 2" if you have owned 3 birds for 5, 4 and 2 years each).
6 months = 0.5 years

☐ Small mammals
→ **Go to 34-36**

34. What type(s) of small mammals do you currently own?
You may select multiple options

☐ Rabbits
☐ Guinea pigs
☐ Hamsters
☐ Gerbils
☐ Mice
☐ Chinchillas

35. How many small mammals do you currently own? ____________
Total number of small mammals owned (regardless of species)

36. For how many years have you owned your current small mammal(s)? _____________
If you own more than 1 small mammals, please list the number of years you own each small mammals individually (for example, type "5, 4, 2" if you have owned 3 small mammals for 5, 4 and 2 years each).
6 months = 0.5 years

37.	For your current pet(s), how much money do you spend on pet care, on average, per month?<br>*Pet care includes non-health-related activities/items: feeding, grooming, toys, apparels.*<br>*in dollars, $*	____________________
38.	For your current pet(s), how much money do you spend on pet health, on average, per month?<br>*Pet health includes health-related activities/items: visits to vets, vaccinations, medications, operations.*<br>*in dollars, $*	____________________
39.	How many times a year do you bring your pet(s) to the veterinary clinic?	____________________
40.	Does your current pet(s) have any medical conditions?	☐ Yes → **Go to 41**<br>☐ No → **Go to 43**
41.	Please state your pet's medical conditions.<br>*For example: Dog 1: Hypertension, Diabetes, Dog 2: Joint pain, Cat 1: Skin condition*	____________________ → **Go to 43**
42.	Have you ever had a pet in the past?<br>*Pets include: dogs, cats, birds, small mammals (e.g. rabbits, guinea pigs, hamsters, gerbils, mice, chinchillas)*	☐ Yes → **Go to 44**<br>☐ No → **Go to 64**
43.	Besides your current pet(s), have you ever had a pet in the past?<br>*Pets include: dogs, cats, birds, small mammals (e.g. rabbits, guinea pigs, hamsters, gerbils, mice, chinchillas)*	☐ Yes → **Go to 44**<br>☐ No → **Go to 55**
44.	At what age did you own your first pet?	____________________

45 What type(s) of pets have you ever owned in the past?
You may select multiple options

☐ Dog<br>→ **Go to 46-47**	46. How many dogs have you ever owned in the past?	____________
	47. For how many years have you owned your previous dog(s)?<br>*If you owned more than 1 dog, please list the number of years you owned each dog individually (for example, type "5, 4, 2" if you have owned 3 dogs for 5, 4 and 2 years each).*<br>*6 months = 0.5 years*	____________
☐ Cat<br>→ **Go to 48-49**	48. How many cats have you ever owned in the past?	____________
	49. For how many years have you owned your previous cat(s)?<br>*If you owned more than 1 cat, please list the number of years you owned each cat individually (for example, type "5, 4, 2" if you have owned 3 cats for 5, 4 and 2 years each).*<br>*6 months = 0.5 years*	____________
☐ Bird<br>→ **Go to 50-51**	50. How many birds have you ever owned in the past?	____________
	51. For how many years have you owned your previous bird(s)?<br>*If you owned more than 1 bird, please list the number of years you owned each bird individually (for example, type "5, 4, 2" if you have owned 3 birds for 5, 4 and 2 years each).*<br>*6 months = 0.5 years*	____________
☐ Small mammals<br>→ **Go to 52-54**	52. What type(s) of small mammals have you ever owned in the past?<br>*You may select multiple options*	☐ Rabbits<br>☐ Guinea pigs<br>☐ Hamsters<br>☐ Gerbils<br>☐ Mice<br>☐ Chinchillas
	53. How many small mammals have you ever owned in the past?<br>*Total number of small mammals owned (regardless of species)*	____________
	54. For how many years have you owned your previous small mammal(s)?<br>*If you owned more than 1 small mammal, please list the number of years you owned each small mammals individually (for example, type "5, 4, 2" if you have owned 3 small mammals for 5, 4 and 2 years each).*<br>*6 months = 0.5 years*	_____________

<u>Section Header: Pet attachment</u> [3,4]
If your household has a pet now → Go to 55
If your household does not have a pet now → Go to 64

55. To what extent do you agree with the following statements regarding your pet(s), in general?

56. I am emotionally close to my pet
☐ 1. Strongly disagree
☐ 2. Disagree
☐ 3. Neutral
☐ 4. Agree
☐ 5. Strongly agree

57. I talk to my pet
☐ 1. Strongly disagree
☐ 2. Disagree
☐ 3. Neutral
☐ 4. Agree
☐ 5. Strongly agree

58. I am not very attached to my pet
☐ 1. Strongly disagree
☐ 2. Disagree
☐ 3. Neutral
☐ 4. Agree
☐ 5. Strongly agree

59. I do not enjoy spending time with my pet
☐ 1. Strongly disagree
☐ 2. Disagree
☐ 3. Neutral
☐ 4. Agree
☐ 5. Strongly agree

60. Often my pet is a nuisance to me
☐ 1. Strongly disagree
☐ 2. Disagree
☐ 3. Neutral
☐ 4. Agree
☐ 5. Strongly agree

61. I consider my pet as a friend
☐ 1. Strongly disagree
☐ 2. Disagree
☐ 3. Neutral
☐ 4. Agree
☐ 5. Strongly agree

62. I have no problem parting with my pet for a long duration
☐ 1. Strongly disagree
☐ 2. Disagree
☐ 3. Neutral
☐ 4. Agree
☐ 5. Strongly agree

63. I view my pet as a member of my family
☐ 1. Strongly disagree
☐ 2. Disagree
☐ 3. Neutral
☐ 4. Agree
☐ 5. Strongly agree

Section Header: General health and mental health [5]

64.	In general, would you say your health is	☐ 5. Excellent<br>☐ 4.Very good<br>☐ 3. Good<br>☐ 2. Fair<br>☐ 1. Poor
65.	Compared to 1 year ago, how would you rate your health in general now?	☐ 5. Much better now than 1 year ago<br>☐ 4. Somewhat better now than 1 year ago<br>☐ 3. About the same<br>☐ 2. Somewhat worse now than 1 year ago<br>☐ 1. Much worse now than 1 year ago

If your household has a pet now → Go to 66
If your household does not have a pet now → Go to 67

66.	Compared to before you had your pet, how would you rate your health in general now?	☐ 5. Much better now than before I had my pet<br>☐ 4. Somewhat better now than before I had my pet<br>☐ 3. About the same<br>☐ 2. Somewhat worse now than before I had my pet<br>☐ 1. Much worse now than before I had my pet

67. During the past 4 weeks, have you had any of the following problems with your work or other regular daily activities as a result of any emotional problems (such as feeling depressed or anxious)?

68.	Cut down the amount of time you spent on work or other activities	☐ Yes<br>☐ No
69.	Accomplished less than you would like	☐ Yes<br>☐ No
70.	Didn't do work or other activities as carefully as usual	☐ Yes<br>☐ No
71.	During the past 4 weeks, to what extent has your physical health or emotional problems interfered with your normal social activities with family, friends, neighbors, or groups?	☐ 1. Not at all<br>☐ 2. Slightly<br>☐ 3. Moderately<br>☐ 4. Severe<br>☐ 5. Very severe

72. These questions are about how you feel and how things have been with you during the past 4 weeks. For each question, please give the one answer that comes closest to the way you have been feeling.

73.	Did you feel full of life?	☐ 1. All of the time<br>☐ 2. Most of the time<br>☐ 3. A good bit of the time<br>☐ 4. Some of the time<br>☐ 5. A little bit of the time<br>☐ 6. None of the time

74.	Have you been a very nervous person?	☐ 1. All of the time<br>☐ 2. Most of the time<br>☐ 3. A good bit of the time<br>☐ 4. Some of the time<br>☐ 5. A little bit of the time<br>☐ 6. None of the time
75.	Have you felt so down that nothing could cheer you up?	☐ 1. All of the time<br>☐ 2. Most of the time<br>☐ 3. A good bit of the time<br>☐ 4. Some of the time<br>☐ 5. A little bit of the time<br>☐ 6. None of the time
76.	Have you felt calm and peaceful?	☐ 1. All of the time<br>☐ 2. Most of the time<br>☐ 3. A good bit of the time<br>☐ 4. Some of the time<br>☐ 5. A little bit of the time<br>☐ 6. None of the time
77.	Did you have a lot of energy?	☐ 1. All of the time<br>☐ 2. Most of the time<br>☐ 3. A good bit of the time<br>☐ 4. Some of the time<br>☐ 5. A little bit of the time<br>☐ 6. None of the time
78.	Have you felt downhearted and blue?	☐ 1. All of the time<br>☐ 2. Most of the time<br>☐ 3. A good bit of the time<br>☐ 4. Some of the time<br>☐ 5. A little bit of the time<br>☐ 6. None of the time
79.	Did you feel worn out?	☐ 1. All of the time<br>☐ 2. Most of the time<br>☐ 3. A good bit of the time<br>☐ 4. Some of the time<br>☐ 5. A little bit of the time<br>☐ 6. None of the time
80.	Have you been a happy person?	☐ 1. All of the time<br>☐ 2. Most of the time<br>☐ 3. A good bit of the time<br>☐ 4. Some of the time<br>☐ 5. A little bit of the time<br>☐ 6. None of the time

81. Did you feel tired?

☐ 1. All of the time
☐ 2. Most of the time
☐ 3. A good bit of the time
☐ 4. Some of the time
☐ 5. A little bit of the time
☐ 6. None of the time

82. During the past 4 weeks, how much of the time has your physical health or emotional problems interfered with your social activities (like visiting with friends, relatives, etc.)?

☐ 1. All of the time
☐ 2. Most of the time
☐ 3. Some of the time
☐ 4. A little bit of the time
☐ 5. None of the time

83. How true or false are each of the following statements for you?

84. I seem to fall sick easier than other people

☐ 1. Definitely true
☐ 2. Mostly true
☐ 3. Don't know
☐ 4. Mostly false
☐ 5. Definitively false

85. I am as healthy as anybody I know

☐ 1. Definitely true
☐ 2. Mostly true
☐ 3. Don't know
☐ 4. Mostly false
☐ 5. Definitively false

86. I expect my health to get worse

☐ 1. Definitely true
☐ 2. Mostly true
☐ 3. Don't know
☐ 4. Mostly false
☐ 5. Definitively false

87. My health is excellent

☐ 1. Definitely true
☐ 2. Mostly true
☐ 3. Don't know
☐ 4. Mostly false
☐ 5. Definitively false

If your household has a pet now → Go to 88
If your household does not have a pet now → Go to 89

88. Compared to before you had your pet, how has your mood changed?

☐ 5. Much better now than before
☐ 4. Somewhat better now than before
☐ 3. About the same
☐ 2. Somewhat worse now than before
☐ 1. Much worse now than before

Section Header: Physical activity [6,7]

89. How many days per week do you engage in **mild-intensity** physical activity?
Mild physical activities are those which allow you to hold a conversation, or even sing while doing them. Examples include: casual walking, taichi, using the elliptical/cross trainer at an easy pace in the gym

☐ 0 → **Go to 92**
☐ 1
☐ 2
☐ 3
☐ 4
☐ 5
☐ 6
☐ 7

90. On average, how many minutes per week do you engage in mild-intensity physical activity?

____

If your household has a dog now → Go to 91
If your household does not have a dog now → Go to 92

91. On average, what percentage (%) of the time spent on mild-intensity physical activity is with your dog?

0 ———————————————— 100
(please mark on the scale from 0 to 100)

92. How many days per week do you engage in **moderate-intensity** physical activity?
Moderate physical activities are those which allow you to hold a short conversation but not sing while doing them. Examples include: jogging, cycling, swimming leisurely, doubles tennis, line-dancing

☐ 0 → **Go to 95**
☐ 1
☐ 2
☐ 3
☐ 4
☐ 5
☐ 6
☐ 7

93. On average, how many minutes per week do you engage in moderate-intensity physical activity?

____

If your household has a dog now → Go to 94
If your household does not have a dog now → Go to 95

94. On average, what percentage (%) of the time spent on moderate-intensity physical activity is with your dog?

0 ———————————————— 100
(please mark on the scale from 0 to 100)

95. How many days a week do you engage in vigorous-intensity physical activity?
Vigorous physical activities are those which do not allow you to hold a short conversation at all while doing them. Examples include: sprinting, sports (soccer, singles tennis, basketball), swimming laps, skipping rope

☐ 0 → **Go to 98 if your household has a pet now**
→ **Go to 99 if your household does not have a pet now**
☐ 1
☐ 2
☐ 3
☐ 4
☐ 5
☐ 6
☐ 7

96. How many minutes per week do you engage in vigorous-intensity physical activity?

____

If your household has a dog now → Go to 97
If your household has a non-dog pet now → Go to 98
If your household does not have a dog now → Go to 99

97.	On average, what percentage (%) of the time spent on vigorous-intensity physical activity is with your dog?	0 ———————————— 100<br>(please mark on the scale from 0 to 100)
98.	Compared to before you had your pet, how has your level of physical activity changed?	☐ 1. Much more active than before I had my pet<br>☐ 2. Somewhat more active than before I had my pet<br>☐ 3. No change<br>☐ 4. Somewhat less active than before I had my pet<br>☐ 5. Much less active than before I had my pet

Section Header: Past medical history

99.	Have you ever been diagnosed with hypertension?<br>*also known as: high blood pressure, high blood*	☐ Yes<br>☐ No
100.	Have you ever been diagnosed with diabetes?<br>*also known as: diabetes mellitus, DM, high sugar, type 2 diabetes mellitus*	☐ Yes<br>☐ No
101.	Have you ever been diagnosed with hyperlipidemia?<br>*also known as: high cholesterol*	☐ Yes<br>☐ No
102.	Have you ever been diagnosed with depression?	☐ Yes<br>☐ No<br>☐ Prefer not to respond
103.	Have you ever been diagnosed with anxiety or other related disorders?<br>*including, but not limited to: generalised anxiety disorder, panic disorder, phobias, post-traumatic stress disorder, acute stress reaction*	☐ Yes<br>☐ No<br>☐ Prefer not to respond
104.	Have you ever been diagnosed with schizophrenia?	☐ Yes<br>☐ No<br>☐ Prefer not to respond

Section Header: Breathing, nose, eye and skin conditions [8,9]

105.	In your entire life, have you had any wheezing or whistling in your chest?	☐ Yes<br>☐ No
**106.**	**In the past 1 year, how often have you had:**	
107.	Any wheezing or whistling in your chest?	☐ Never<br>☐ Rarely<br>☐ Sometimes<br>☐ Most days<br>☐ Everyday

108. Any tightness of chest?

☐ Never
☐ Rarely
☐ Sometimes
☐ Most days
☐ Everyday

109. Any shortness of breath at rest?

☐ Never
☐ Rarely
☐ Sometimes
☐ Most days
☐ Everyday

110. Any shortness of breath when doing strenuous activity?

☐ Never
☐ Rarely
☐ Sometimes
☐ Most days
☐ Everyday

111. Been disturbed from sleep, due to tightness of chest or shortness of breath?

☐ Never
☐ Rarely
☐ Sometimes
☐ Most days
☐ Everyday

112. To use medications to relieve any of the above symptoms?

☐ Never
☐ Rarely
☐ Sometimes
☐ Most days
☐ Everyday

For 107-111, if you answered 'Never' to <u>all</u> → Go to 114
Otherwise → Go to 113

113. Difficulties carrying out day-to-day activities because of any of the above symptoms?

☐ Never
☐ Rarely
☐ Sometimes
☐ Most days
☐ Everyday

114. Have you ever had asthma?

☐ Yes → **Go to 115**
☐ No → **Go to 118 if you answered 'Yes' to 105**
→ **Go to 121 if you answered 'No' to 105**

115. Was your asthma confirmed by the doctor?

☐ Yes → **Go to 116**
☐ No → **Go to 117**

116. Have you ever been prescribed medications for your asthma?

☐ Yes
☐ No

117. How many asthma attacks have you experienced in the past 1 year?
enter 0 if there were none in the past 1 year

____

For 105 and 114, if you answered 'No' to <u>both</u> → Go to 121
Otherwise → Go to 118

118. In the past 1 year, how have your breathing symptoms changed?
- ☐ 1. Much worse
- ☐ 2. Slightly worse
- ☐ 3. No change
- ☐ 4. Slightly better
- ☐ 5. Much better

If your household has/had a pet now or in the past → Go to 119
If your household never had a pet → Go to 121

119. When you first got your pet, how did your breathing symptoms change?
- ☐ 1. Much worse
- ☐ 2. Slightly worse
- ☐ 3. No change
- ☐ 4. Slightly better
- ☐ 5. Much better

If your household had a pet in the past → Go to 120
If your household did not have a pet in the past → Go to 121

120. When you no longer had your pet, how did your breathing symptoms change?
- ☐ 1. Much worse
- ☐ 2. Slightly worse
- ☐ 3. No change
- ☐ 4. Slightly better
- ☐ 5. Much better

121. In your entire life, have you had any problems with sneezing, runny or blocked nose when you were NOT having a cold or flu?
- ☐ Yes
- ☐ No

122. In the past 1 year, how often have you had:

123. Problems with sneezing, runny, or blocked nose when you were not down with a cold or flu?
- ☐ Never
- ☐ Rarely
- ☐ Sometimes
- ☐ Most days
- ☐ Everyday

124. Itchy or red eyes?
- ☐ Never
- ☐ Rarely
- ☐ Sometimes
- ☐ Most days
- ☐ Everyday

125. To use medications to relieve any of the above symptoms?
- ☐ Never
- ☐ Rarely
- ☐ Sometimes
- ☐ Most days
- ☐ Everyday

For 123-124, if you answered 'Never' to <u>both</u> → Go to 127
Otherwise → Go to 126

126. Difficulties carrying out day-to-day activities because of any of the above symptoms?

☐ Never
☐ Rarely
☐ Sometimes
☐ Most days
☐ Everyday

127. Have you ever had morning sinus or allergic/sensitive nose?

☐ Yes → **Go to 128**
☐ No → **Go to 130 if you answered 'Yes' to 121**
→ **Go to 133 if you answered 'No' to 121**

128. Have you ever been diagnosed by a doctor to have allergic rhinitis (AR)?

☐ Yes → **Go to 129**
☐ No → **Go to 130**

129. Have you ever been prescribed medications for your allergic rhinitis (AR)?

☐ Yes
☐ No

For 121 and 127, if you answered 'No' to both → Go to 133
Otherwise → Go to 130

130. In the past 1 year, how have your symptoms changed?

☐ 1. Much worse
☐ 2. Slightly worse
☐ 3. No change
☐ 4. Slightly better
☐ 5. Much better

If your household has/had a pet now or in the past → Go to 131
If your household never had a pet → Go to 133

131. When you first got your pet, how did your symptoms change?

☐ 1. Much worse
☐ 2. Slightly worse
☐ 3. No change
☐ 4. Slightly better
☐ 5. Much better

If your household had a pet in the past → Go to 132
If you household did not have a pet in the past → Go to 133

132. When you no longer had your pet, how did your symptoms change?

☐ 1. Much worse
☐ 2. Slightly worse
☐ 3. No change
☐ 4. Slightly better
☐ 5. Much better

133. In your entire life, have you had any problems with an itchy rash that comes and goes?

☐ Yes
☐ No

134. In the past 1 year, have often have you had:

135. An itchy rash that comes and goes, and affected either one of the following: fold of elbows, behind knees, face, neck?

☐ Never
☐ Rarely
☐ Sometimes
☐ Most days
☐ Everyday

136. To use medications to relieve the above rash?

☐ Never
☐ Rarely
☐ Sometimes
☐ Most days
☐ Everyday

For 135, if you answered 'Never' → Go to 138
Otherwise → Go to 137

137. Difficulties carrying out day-to-day activities due to any of the above symptoms?

☐ Never
☐ Rarely
☐ Sometimes
☐ Most days
☐ Everyday

138. Have you ever had eczema?

☐ Yes → **Go to 139**
☐ No → **Go to 141 if you answered 'Yes' to 133**
→ END OF QUESTIONNAIRE if you answered 'No' to 133

139. Was your eczema confirmed by the doctor?

☐ Yes → **Go to 140**
☐ No → **Go to 141**

140. Have you ever been prescribed medications for your eczema?

☐ Yes
☐ No

For 133 and 138, if you answered 'No' to <u>both</u> → END OF QUESTIONNAIRE
Otherwise → Go to 141

141. In the past 1 year, how have your symptoms changed?

☐ 1. Much worse
☐ 2. Slightly worse
☐ 3. No change
☐ 4. Slightly better
☐ 5. Much better

If your household has/had a pet now or in the past → Go to 142
If your household never had a pet → END OF QUESTIONNAIRE

142. When you first got your pet, how did your symptoms change?

☐ 1. Much worse
☐ 2. Slightly worse
☐ 3. No change
☐ 4. Slightly better
☐ 5. Much better

If your household had a pet in the past → Go to 143
If you household did not have a pet in the past → END OF QUESTIONNAIRE

143. When you no longer had your pet, how did your symptoms change?

☐ 1. Much worse
☐ 2. Slightly worse
☐ 3. No change
☐ 4. Slightly better
☐ 5. Much better

END OF QUESTIONNAIRE

Statistical analysis

Supplementary Table S1. Final propensity score model listing factors which predict assignment to pet ownership over non-ownership

Covariate	OR (95% CI)	*P*-value
**Age category**		
21-30 y	1 (ref)	(ref)
31-40 y	**2.154 (1.375-3.373)**	**0.0008**
41-50 y	**1.780 (1.089-2.911)**	**0.0214**
51-64 y	0.919 (0.544-1.554)	0.7531
**Race**		
Chinese	1 (ref)	(ref)
Malay	**2.883 (1.539-5.399)**	**0.0009**
Indian	0.693 (0.350-1.371)	0.2918
Others	1.758 (0.807-3.827)	0.1554
**Marriage##Housing**		
Married & 1-5 room HDB	1 (ref)	(ref)
Married & (Exec. HDB or Condo)	0.923 (0.547-1.589)	0.7738
Married & Landed property	1.775 (0.711-4.433)	0.2189
Single & 1-5 room HDB	0.901 (0.564-1.439)	0.6630
Single & (Exec. HDB or Condo)	0.955 (0.544-1.680)	0.8747
Single & Landed property	**2.536 (1.276-5.040)**	**0.0079**
**Female gender**	**1.697 (1.177-2.448)**	**0.0046**
**Past pet owner**	**2.619 (1.924-3.565)**	**<0.0001**

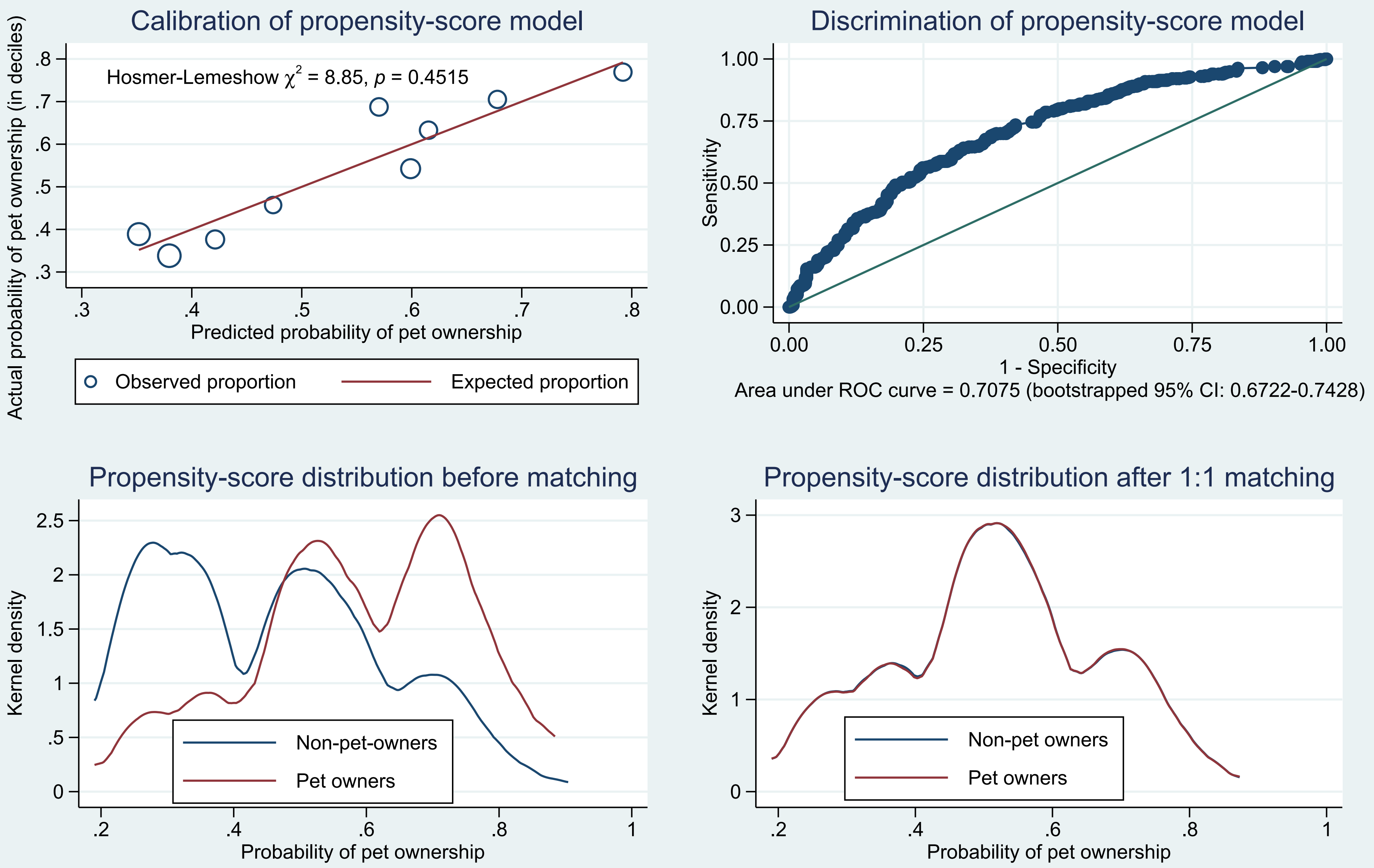

Supplementary Figure S1. Propensity-score model and matching diagnostics. Propensity-scores (i.e., conditional probability of pet ownership) were estimated using logistic regression modelling of baseline demographics and other relevant covariates, and showed good calibration and discrimination. Propensity-scores between pet-owners *vs* non-pet-owners were well-balanced after 1:1 greedy matching using a caliper of 0.25 * standard deviation of the linear predictor (i.e., logit).

Supplementary Table S2. Citation of existing literature studying relationship between chosen outcome measures and categorical moderator variables.

	Physical Activity Levels	Mental Well-being	Atopic Conditions
**DEMOGRAPHICAL**			
Age	P1	M1	A1
Gender	P2	M2	A2
Race	P3	M3	A3
Marriage	P4	M4	A4
Housing	P5	M5	A5
Education	P6	M6	A6
Employment	P7	M7	A7
Income	P8	M8	A8
**PET OWNERSHIP TYPE**			
Main caregiver vs Non-main caregiver	P9	M9	-
Pet type	P10	M10	A10
Pet Attachment Survey Score	P11	M11	-

P1 Increased participation in physical activity with increasing age

Canizares, M., & Badley, E. M. (2018). Generational differences in patterns of physical activities over time in the Canadian population: an age-period-cohort analysis. BMC Public Health, 18(1). doi: 10.1186/s12889-018-5189-z

Levels and frequencies of physical activity decreased with age.

Li, W., Churchill, L., Procter-Gray, E., Kane, K., Cheng, J., Clarke, A., & Ockene, J. (2017). Sex Differences In Physical Activity Among Older Adults Living In Urban And Rural Neighborhoods. Innovation in Aging, 1(suppl_1), 1092–1092. doi: 10.1093/geroni/igx004.4005

P2 Adolescent males participated in more physical activity

Tiggelman, D., Monique O. M. Van De Ven, Schayck, O. C. P. V., & Engels, R. C. M. E. (2014). Moderating effect of gender on the prospective relation of physical activity with psychosocial outcomes and asthma control in adolescents: a longitudinal study. Journal of Asthma, 51(10), 1049–1054. doi: 10.3109/02770903.2014.941475

Males clocked 16% higher step counts, and had higher self-reported frequency of exercise as compared to women.

Li, W., Churchill, L., Procter-Gray, E., Kane, K., Cheng, J., Clarke, A., & Ockene, J. (2017). Sex Differences In Physical Activity Among Older Adults Living In Urban And Rural Neighborhoods. Innovation in Aging, 1(suppl_1), 1092–1092. doi: 10.1093/geroni/igx004.4005

P3 Chinese participants engaged in less physical activity than Malays and other races in Malaysia.

Lian, T. C., Bonn, G., Han, Y. S., Choo, Y. C., & Piau, W. C. (2016). Physical Activity and Its Correlates among Adults in Malaysia: A Cross-Sectional Descriptive Study. Plos One, 11(6). doi: 10.1371/journal.pone.0157730

P4 Time spent in moderate-vigorous physical activity declined after marriage.

Miller, J., Nelson, T., Barr-Anderson, D. J., Christoph, M. J., Winkler, M., & Neumark-Sztainer, D. (2019). Life Events and Longitudinal Effects on Physical Activity: Adolescence to Adulthood. Medicine and science in sports and exercise, 51(4), 663–670.

P5 Increased leisure time sitting was associated with a decrease in household size.

Madina, S., Torben, J., Rikke, J., Allan, L., & Mette, A. (2015). The influence of housing characteristics on leisure-time sitting. A prospective cohort study in Danish adults. Preventive medicine. 81. 10.1016/j.ypmed.2015.08.001.

P6 Negative relationship between education level and physical activity

Tam, C. L., Bonn, G., Yeoh, S. H., Choo, Y., & Wong, C. P. (2016) Physical Activity and Its

Correlates among Adults in Malaysia: A Cross Sectional Descriptive Study. PLoS ONE 11(6): e0157730. doi:10.1371/journal.pone.0157730

P7 Paid workers had significantly higher levels of physical activity
Tan, M. E., Sagayadevan, V., Abdin, E., Picco, L., Vaingankar, J., Chong, S. A., & Subramaniam, M. (2017). Employment status among the Singapore elderly and its correlates. Psychogeriatrics : the official journal of the Japanese Psychogeriatric Society, 17(3), 155–163

Participants in low-level occupations were more active and less sedentary than non-employed
Pulakka, A., Stenholm, S., Bosma, H., Schaper, N. C., Savelberg, H., Stehouwer, C., van der Kallen, C., Dagnelie, P. C., Sep, S., & Koster, A. (2018). Association Between Employment Status and Objectively Measured Physical Activity and Sedentary Behavior-The Maastricht Study. Journal of occupational and environmental medicine, 60(4), 309–315.

P8 Leisure-time physical activity, and specifically vigorous leisure-time physical activity, is less prevalent while occupational physical activity is more prevalent among people with lower socioeconomic position.
Beenackers, M. A., Kamphuis, C. B. M., Giskes, K., Brug. J., Kunst. A. E., Burdorf. A., & van Lenthe, F. J. (2012) Socioeconomic inequalities in occupational, leisure-time, and transport related physical activity among European adults: a systematic review. Int J Behav Nutr Phys Act. 2012;9:116

Being retired and reporting low income were significantly associated with higher odds of sedentary behavior as compared to students and patients with high income status.
Konevic, S., Martinovic, J., & Djonovic, N. (2015). Association of Socioeconomic Factors and Sedentary Lifestyle in Belgrade's Suburb, Working Class Community. Iranian journal of public health, 44(8), 1053–1060.

P9 Walking activity was greater for current and past dog/cat owners than for never owners
Taniguchi, Y., Seino, S., Nishi, M., Tomine, Y., Tanaka, I., Yokoyama, Y., Armano, H., Kitamura, A., & Shinkai, S. (2018) Physical, social, and psychological characteristics of community-dwelling elderly Japanese dog and cat owners. PLoS ONE 13(11): e0206399.

P10 Dog ownership is associated with more light physical activity as compared to cat owners.
Taniguchi, Y., Seino, S., Nishi, M., Tomine, Y., Tanaka, I., Yokoyama, Y., Armano, H., Kitamura, A., & Shinkai, S. (2018) Physical, social, and psychological characteristics of community-dwelling elderly Japanese dog and cat owners. PLoS ONE 13(11): e0206399.

Current pet-owners reported more physical activity than did previous pet owners.
Chowdhury, E. K., Nelson, M. R., Jennings, G. L. R., Wing, L. M. H., & Reid, C. M. (2017). Pet ownership and survival in the elderly hypertensive population. Journal of Hypertension, 35(4), 769–775. doi:10.1097/hjh.0000000000001214

P11 Higher levels of child attachment to a pet dog were associated with more time spent being active with a pet dog
Gadomski, A. M., Scribani, M. B., Krupa, N., and Jenkins, P. (2017) Pet dogs and child physical activity: the role of child–dog attachment. Pediatric Obesity, 12: e37– e40. doi: 10.1111/ijpo.12156.

M1 Age of onset for most severe psychiatric disorders peaks in early adulthood with the majority of disorders emerging by the age of 25.
Kiely, K. M., Brady, B., & Byles, J. (2019). Gender, mental health and ageing. Maturitas, 129, 76–84. doi: 10.1016/j.maturitas.2019.09.004

Mental health conditions such as dementia and depression are more common in later life.
Almeida, O. P., Norman, P., Hankey, G., Jamrozik, K., & Flicker, L. (2006). Successful Mental Health Aging: Results From a Longitudinal Study of Older Australian Men. The American Journal of Geriatric Psychiatry, 14(1), 27–35. doi: 10.1097/01.jgp.0000192486.20308.42

M2 Women have a higher lifetime prevalence of mood and anxiety disorders whereas men are overrepresented with externalising disorders (e.g. substance use disorders) and suicide mortality
Kiely, K. M., Brady, B., & Byles, J. (2019). Gender, mental health and ageing. Maturitas, 129, 76–84. doi: 10.1016/j.maturitas.2019.09.004

Low social well-being was significantly higher in women than in men, which in turn i associated with depression, anxiety and sleeping problems.

Lukaschek, K., Vanajan, A., Johar, H., Weiland, N., & Ladwig, K.-H. (2017). "In the mood for ageing": determinants of subjective well-being in older men and women of the population-based KORA-Age study. BMC Geriatrics, 17(1). doi: 10.1186/s12877-017-0513-5

M3 Lifetime prevalence of MDD was significantly higher among the Indians than the Chinese and Malays
Chong, S. A., Vaingankar, J., Abdin, E., & Subramaniam, M. (2012). The prevalence and impact of major depressive disorder among Chinese, Malays and Indians in an Asian multi-racial population. Journal of Affective Disorders, 138(1-2), 128–136. doi: 10.1016/j.jad.2011.11.038

Indians exhibited higher rates of depression as compared to chinese and Malays. Minority groups are known to experience higher anxiety and depression rates due to multiple stressors.
Ganasegeran, K., Renganathan, P., Manaf, R. A., & Al-Dubai, S. A. R. (2014). Factors associated with anxiety and depression among type 2 diabetes outpatients in Malaysia: a descriptive cross-sectional single-centre study. BMJ Open, 4(4). doi: 10.1136/bmjopen-2014-004794

M4 Decline in mental health for men who were separated or widowed, as compared to men who remained married.
Similar declines in mental health were found for women who separated or became widowed.
Hewitt, B., Turrell, G., & Giskes, K. (2010). Marital loss, mental health and the role of perceived social support: findings from six waves of an Australian population based panel study. Journal of Epidemiology and Community Health, 66(4), 308–314.

Compared to married elderly people, the widowed, divorced and never-married people had a higher risk of depression.
Yan, X.-Y., Huang, S.-M., Huang, C.-Q., Wu, W.-H., & Qin, Y. (2011). Marital Status and Risk for Late Life Depression: A Meta-Analysis of the Published Literature. Journal of International Medical Research, 39(4), 1142–1154. doi: 10.1177/147323001103900402

M5 Variables that had significant association with depressive symptoms included the type of housing (squatting, flat/apartment, semi-detached house, bungalow).
Ang, L., Suzaily. W., Fairuz. N., Fairuz. A. R., Helmy. H., & Rosliwati. Y. (2018). Depressive Symptoms among Adolescents in Kuching, Malaysia: prevalence and associated factors. Pediatrics International. 61. 10.1111/ped.13778.

M6 Compared with respondents with college and higher level of education, respondents with less than high school levels of education reported significantly worse mental health.
Wei. Z., Qi. C., Hamilton. M., Lali. L., & Shirley. F., (2010) Predictors of mental and physical health: Individual and neighborhood levels of education, social well-being, and ethnicity. Health & place. 17. 10.1016/j.healthplace.2010.10.008.

Higher education was associated with good mental health, whereas poor education is associated with increased risk in dementia and cognitive impairment.
Almeida, O. P., Norman, P., Hankey, G., Jamrozik, K., & Flicker, L. (2006). Successful Mental Health Aging: Results From a Longitudinal Study of Older Australian Men. The American Journal of Geriatric Psychiatry, 14(1), 27–35. doi: 10.1097/01.jgp.0000192486.20308.42

M7 Temporary workers, first-job seekers and unemployed individuals are worse off in terms of physical and mental health than permanent employees.
Minelli, L., Pigini, C., Chiavarini, M., & Bartolucci, F. (2014). Employment status and perceived health condition: longitudinal data from Italy. BMC Public Health, 14, 946.

M8 Lower income at the household level itself had an adverse effect on mental health.
Fujita, M., Nagashima, K., Takahashi, S., & Hata, A. (2019). Inequality within a community at the neighborhood level and the incidence of mood disorders in Japan: a multilevel analysis. Social Psychiatry and Psychiatric Epidemiology, 54(9), 1125–1131. doi: 10.1007/s00127-019-01687-w

M9 Loneliness and social isolation seems to be ameliorated by pet ownership.
Needell, N.J., & Mehta-Naik, N. (2016) Is Pet Ownership Helpful in Reducing the Risk and Severity of Geriatric Depression? Geriatrics 2016, 1, 24.

M10 Cat ownership was related to fewer episodes of bad mood.
Taniguchi, Y., Seino, S., Nishi, M., Tomine, Y., Tanaka, I., Yokoyama, Y., Armano, H., Kitamura, A., & Shinkai, S. (2018) Physical, social, and psychological characteristics of community-dwelling elderly Japanese dog and cat owners. PLoS ONE 13(11): e0206399.

M11 The degree of attachment to dogs showed the highest coefficient value in Sense of Life Worth Living scale

Nagasawa, M., & Ohta, M. (2010). The influence of dog ownership in childhood on the sociality of elderly Japanese men. Animal science journal = Nihon chikusan Gakkaiho, 81 3, 377-83.

A1 Peak age for positive patch test reactions were 30-49 (females) and 70-79 (males); elderly patients were also more likely to have multiple allergies.
Foley, P., Zuo, Y., Plunkett, A., Merlin, K., & Marks, R. (2003). The Frequency of Common Skin Conditions in Preschool-aged Children in Australia. Archives of Dermatology, 139(3). doi: 10.1001/archderm.139.3.318

Prevalence of allergic rhinoconjunctivitis peaked at age 10, prevalence of atopic dermatitis was highest in younger children, and there was a strong correlation between age and prevalence of asthma.
Futamura, M., Ohya, Y., Akashi, M., Adachi, Y., Odajima, H., Akiyama, K., & Akasawa, A. (2011). Age-related Prevalence of Allergic Diseases in Tokyo Schoolchildren. Allergology International, 60(4), 509–515. doi: 10.2332/allergolint.10-oa-0293

A2 Females had a lower peak age for positive patch test reactions (30-49) as compared to males (70-79).
Foley, P., Zuo, Y., Plunkett, A., Merlin, K., & Marks, R. (2003). The Frequency of Common Skin Conditions in Preschool-aged Children in Australia. Archives of Dermatology, 139(3). doi: 10.1001/archderm.139.3.318

Significantly higher rates of asthma were seen in boys as compared to girls.
Futamura, M., Ohya, Y., Akashi, M., Adachi, Y., Odajima, H., Akiyama, K., & Akasawa, A. (2011). Age-related Prevalence of Allergic Diseases in Tokyo Schoolchildren. Allergology International, 60(4), 509–515. doi: 10.2332/allergolint.10-oa-0293

A3 Malays and Indians had higher rates of asthma mortality and morbidity than the Chinese.
Ng, T. P. (1999). Adult Asthma Prevalence, Morbidity and Mortality and Their Relationships with Environmental and Medical Care Factors in Singapore. Asian Pacific Journal of Allergy and Immunology, 127–135.

A4 Atopic dermatitis was associated with being divorced, separated and unmarried adults.
Hua, T., & Silverberg, J. I. (2018). Atopic dermatitis in US adults: Epidemiology, association with marital status, and atopy. Annals of Allergy, Asthma & Immunology , 121(5), 622–624.

Higher prevalence of asthma amongst single, divorced, or widowed persons than for those that were married, in a civil union or cohabitating.
Solet, J.-L., Raherison-Semjen, C., Mariotti, E., Strat, Y. L., Gallay, A., Bertrand, E., Jahaly, N. & Filleul, L. (2019). A cross sectional survey to estimate prevalence and associated factors of asthma on Reunion Island, Indian Ocean. BMC Public Health, 19(1). doi: 10.1186/s12889-019-7031-7

A5 High-rise apartments were associated with the existence of cockroach allergens.
Rosenfeld, L., Chew, G. L., Rudd, R., Emmons, K., Acosta, L., Perzanowski, M., & Acevedo-García, D. (2011). Are building-level characteristics associated with indoor allergens in the household?. Journal of urban health : bulletin of the New York Academy of Medicine, 88(1), 14–29.

A6 Subjects in the low occupational class and education group had higher mean asthma scores than those in higher socioeconomic groups.
Ellison-Loschmann, L., Sunyer, J., Plana, E., Pearce, N., Zock, J. P., Jarvis, D., Janson, C., Antó, J. M., Kogevinas, M., & European Community Respiratory Health Survey (2007). Socioeconomic status, asthma and chronic bronchitis in a large community-based study. The European respiratory journal, 29(5), 897–905.

A7 A lower proportion of subjects that were currently employed reported respiratory symptoms as compared to those not in current employment.
Senthilselvan, A., Coonghe, W., & Beach, J. (2020). Respiratory health, occupation and the healthy worker effect. Occupational medicine (Oxford, England), 70(3), 191–199.

A8 A lower socioeconomic status was associated with worse asthma control.
Bacon, S.L., Bouchard, A., Loucks, E.B., & Lavoie, K. L. (2009) Individual-level socioeconomic status is associated with worse asthma morbidity in patients with asthma. Respir Res 10, 125 (2009).

A lower socioeconomic position was associated with higher prevalence of asthma.
Uphoff, E., Cabieses, B., Pinart, M., Valdés, M., Antó, J. M., & Wright, J. (2015). A systematic review of socioeconomic position in relation to asthma and allergic diseases. European Respiratory Journal, 46(2), 364-374.

Severe eczema was associated with lower socioeconomic status.
Silverberg, J. I., & Simpson, E. L. (2014). Associations of childhood eczema severity: a US population-based study. Dermatitis : contact, atopic, occupational, drug, 25(3), 107–114.

A10 Cat owners was associated with an increase in asthma prevalence, while dog owners were more likely to have lower spirometry values.
Simoneti CS, Ferraz E, Menezes MB, Icuma TR and Vianna EO (2018) Cat ownership is associated with increased asthma prevalence and dog ownership with decreased spirometry values. Braz J Med Biol Res; doi: 10.1590/1414-431x2-187558

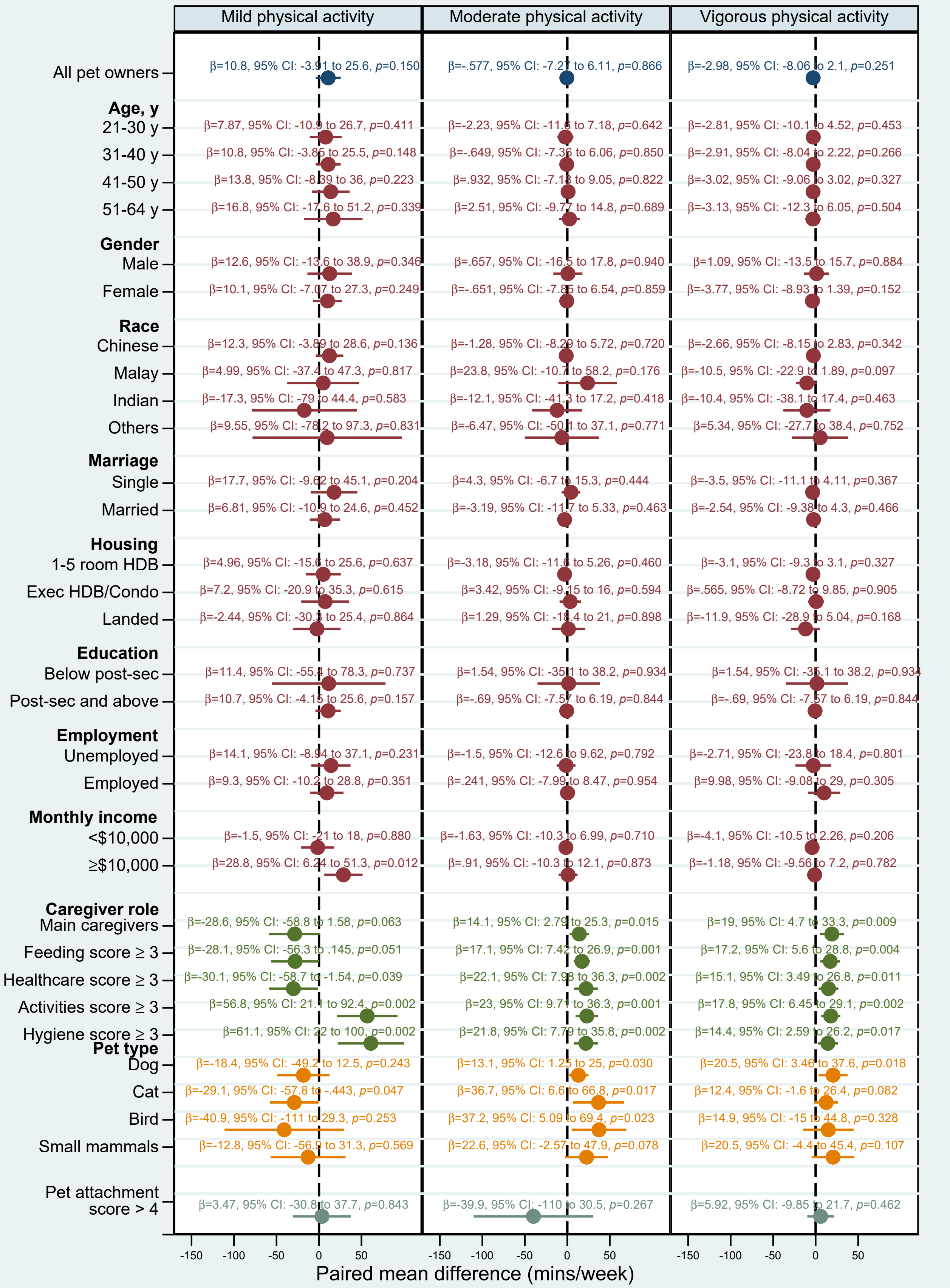

Supplementary Figure S2. Propensity-score-matched comparison of weekly physical activity levels between pet-owners vs non-pet-owners in the full matched set as well as selected subgroups. Subgroup-specific effects were computed as marginal contrasts by specifying a full factorial interaction between pet ownership and the relevant covariate.

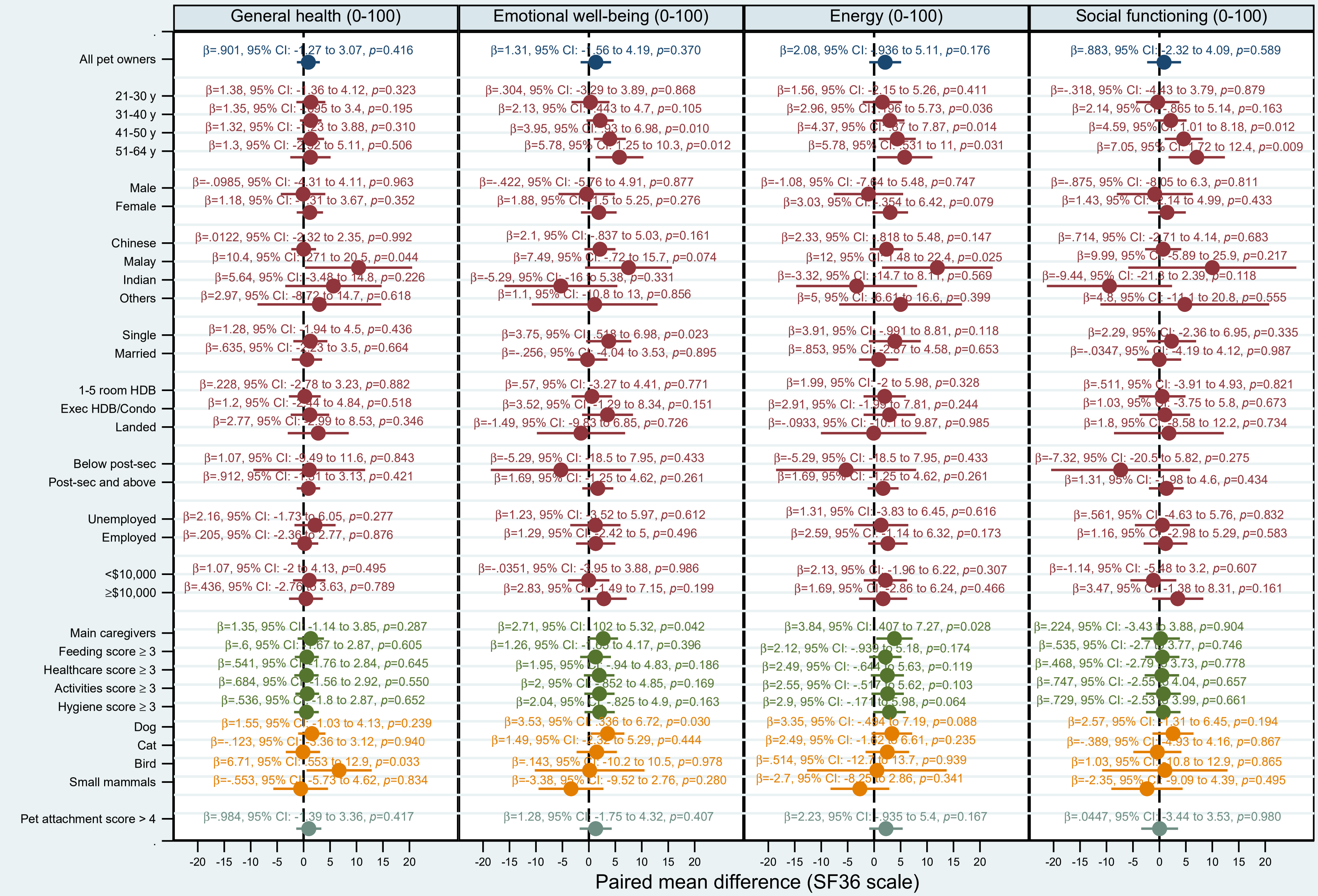

Supplementary Figure S3. Propensity-score-matched comparison of SF36 subjective domain scores between pet-owners vs non-pet-owners in the full matched set as well as selected subgroups. Subgroup-specific effects were computed as marginal contrasts by specifying a full factorial interaction between pet ownership and the relevant covariate.

References

1 Agri-Food & Veterinary Authority of Singapore (AVA). *Code of Animal Welfare (for the Pet Industry).* <https://www.nparks.gov.sg/avs/-/media/avs_-caw-pet-industry-full-(eng).pdf> (2016).

2 Agri-Food & Veterinary Authority of Singapore (AVA). *Code of Animal Welfare (for Pet Owners).* <https://www.nparks.gov.sg/avs/-/media/avs_-caw_booklet-(eng).pdf> (2017).

3 Holcomb, R., Williams, R. C. & Richards, P. S. The elements of attachment: Relationship maintenance and intimacy. *Journal of the Delta Society* **2**, 28-34 (1985).

4 Zilcha-Mano, S., Mikulincer, M. & Shaver, P. R. An attachment perspective on human–pet relationships: Conceptualization and assessment of pet attachment orientations. *Journal of Research in Personality* **45**, 345-357, doi:https://doi.org/10.1016/j.jrp.2011.04.001 (2011).

5 Hays, R. D., Sherbourne, C. D. & Mazel, R. M. The RAND 36-item health survey 1.0. *Health Economics* **2**, 217-227, doi:10.1002/hec.4730020305 (1993).

6 Epidemiology & Disease Control Division, Ministry of Health, Singapore. *National Health Survey 2010.*, <https://www.moh.gov.sg/docs/librariesprovider5/resources-statistics/reports/nhs2010---low-res.pdf?sfvrsn=e54926c6_0> (2011).

7 Health Promotion Board (HPB), Singapore. *National Physical Activity Guidelines: Professional Guide.*, <https://www.healthhub.sg/sites/assets/Assets/PDFs/HPB/PhysicalActivityPDFs/NPAG_Professional_Guide.pdf> (2011).

8 The European Community Respiratory Health Survey II Steering Committee. The European Community Respiratory Health Survey II. *European Respiratory Journal* **20**, 1071-1079, doi:10.1183/09031936.02.00046802 (2002).

9 Asher, M. *et al.* International Study of Asthma and Allergies in Childhood (ISAAC): rationale and methods. *European Respiratory Journal* **8**, 483-491 (1995).
